# Polymorph Screening and Investigation of Charge Transport of ditBuC6-BTBT

**DOI:** 10.1021/acs.cgd.5c00046

**Published:** 2025-06-03

**Authors:** Priya Pandey, Federico Modesti, Nemo McIntosh, Christian Ruzié, Nicholas Turetta, Lamiaa Fijahi, Massimiliano Remigio, Guillaume Schweicher, Yves Henri Geerts, Marta Mas-Torrent, Peter Erk, Jérôme Cornil, Paolo Samorì, Enrico Modena, Lucia Maini

**Affiliations:** † PolyCrystalLine SPA, Via Della Cooperazione, Medicina, 29 40059 Bologna, Italy; ‡ Dipartimento di Chimica “G. Ciamician”, via Selmi 2—Università di Bologna, I-40126 Bologna, Italy; § Laboratoire de Chimie des Polymères, Faculté des Sciences, 26659 Université Libre de Bruxelles (ULB), Boulevard du Triomphe, 1050 Bruxelles, Belgium; ∥ 5184 BASF SE, Carl-Bosch-Straße 38, 67063 Ludwigshafen am Rhein, Germany; ⊥ Laboratory for Chemistry of Novel Materials, 54521 University of Mons, 7000 Mons, Belgium; # 27083 University of Strasbourg, CNRS, ISIS UMR 7006, 8 Alleé Gaspard Monge, F-67000 Strasbourg, France; ∇ 54449 Institut de Ciència de Materials de Barcelona (ICMAB-CSIC), Campus de la UAB, 08193 Bellaterra, Spain; ○ International Solvay Institutes, Université Libre de Bruxelles (ULB), CP 231 Boulevard du Triomphe, 1050 Bruxelles, Belgium; ◆ WEL Research Institute, avenue Pasteur, 6, 1300 Wavre, Belgium; ¶ rConTec GmbH, Roter-Turm-Weg 3, 67157 Wachenheim an der Weinstraße, Germany

## Abstract

In this study, we investigate the relationship between the polymorphism and crystallographic parameters and the charge transport properties achieved through the fabrication of organic field-effect transistors (OFETs) based on a novel molecular semiconductor, i.e., 2,7-bis­(7,7-dimethyloctyl)­benzo­[*b*]­benzo­[4,5]­thieno­[2,3-*d*]­thiophene (ditBuC6-BTBT). Four polymorphs of ditBuC6-BTBT were identified: three observed at room temperature (Forms I, Ia, and II), and one appearing above 100 °C (Form III). While cell parameters were measured for all forms, full crystal structures were determined only for Forms Ia and II. Although a direct correlation between molecular packing and charge transport properties could not be established from the present study, the structural analysis of the polymorphs contributes to a broader understanding of the packing motifs in ditBuC6-BTBT. A meticulous examination of the minute discrepancies in the powder patterns substantiated the existence of both the metastable Form I and Form Ia, which became more difficult to isolate due to unintentional seeding of the thermodynamically stable Form II. Nonequilibrium crystallization techniques utilizing thermal gradient and bar-assisted meniscus shearing methods were explored to enhance control over polymorph selection. The intrinsic charge transport properties ruled by the overlap of the frontier orbitals were studied by computing the transfer integrals. Optimized devices fabricated by depositing thin films by solution shearing and vacuum evaporation led to field-effect mobility in the linear regime of ca. 0.05 cm^2^ V^–1^ s^–1^. The observed device performances were interpreted as a result of the combined effects of crystal packing features, ionization potential values, and polymorphic coexistence, highlighting the challenges in deriving clear structure–property correlations and underscoring the complexities in achieving high-performance organic electronics with this material.

## Graphical abstract

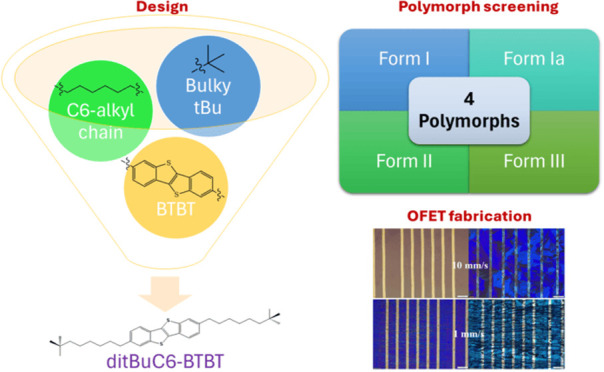

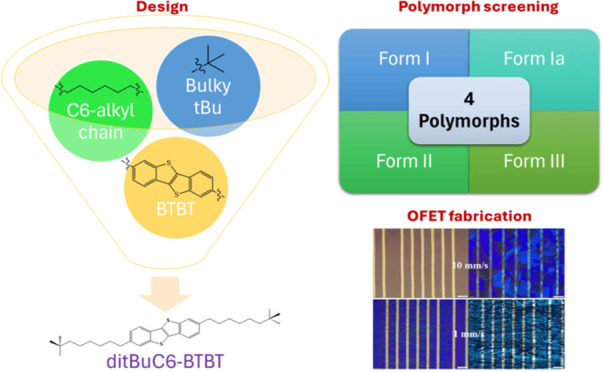

## Introduction

Highlighting the critical role of polymorphism (i.e., the ability of a compound to exhibit more than one molecular packing motif^1^) underscores how the organization of molecules significantly influences and dictates the process of charge transport in organic semiconductors (OSCs) and other physical properties.^2–6^ Multiple factors can guide the formation of polymorphs by mainly influencing the nucleation and growth, which are the two critical processes underlying the formation of polymorphs, and both depend on thermodynamic and kinetic aspects. The crystalline form that is obtained is therefore not necessarily solely the thermodynamic form, but it is possible to obtain metastable kinetic forms that may exist for a few seconds or years; furthermore, in some cases, the thermodynamic form is isolated years later than the first synthesis of the molecule.^7^ It is also possible that an easily obtained metastable form becomes difficult to reproduce when the thermodynamically stable polymorph is formed.^8^ Another crucial point is the occurrence of concomitant polymorphs,^9^ which may lead to difficulties in isolating crystal forms. There are several ways to tackle these issues and control the polymorph formation: (i) solvent-induced polymorph selectivity: the solvent may have a profound impact on the structure of nuclei through solvent–molecule interactions that govern the formation of a particular structure;^10^ (ii) temperature control: thermotropic polymorphs can be identified by investigating a wide range of temperatures;^11,12^ (iii) deposition control (for thin films):^13,14^ nonconventional crystallization techniques (like directional crystallization and solution shearing on substrates) can be explored for polymorph investigation, as a wide range of parameters can be optimized to drive the crystallization process toward one specific polymorph;^11^ (iv) postdeposition control: thermal- and solvent-vapor annealing can be used for increasing crystallinity and sometimes to alter the molecular packing in the OSC films.^15–18^

In organic electronics, the polymorphism of OSCs may have severe consequences on charge carrier mobility, providing an opportunity to understand the importance of molecular packing on charge transport.^19^ Polymorphism is not only critical for understanding structure–property relationships but also offers opportunities for technological applications. For instance, in materials like rubrene^20,21^ and TIPS-pentacene,^22,23^ different polymorphs have been shown to strongly influence the charge transport properties and overall device performance, highlighting the critical need to control and understand polymorphism in organic semiconductors. The electronic properties can be directly related to the structural differences.^15,24,25^ For example, it has been reported that enhancing molecular order through optimized crystallization can lead to a significant increase in mobility, highlighting the strong link between crystal packing and transport properties.^26^ Within this context, studying substrate-induced and thin-film polymorphism is essential as interfacial phases can exhibit a molecular packing different from bulk phases.^11,27^ This is even more important in the case of a transistor, because the zone actively engaged in charge transport typically encompasses the first few monolayers at the interface with the dielectric. Aiming to control polymorphism in thin films, we explored different deposition techniques, such as directional crystallization using thermal gradient and solution shearing methods, on glass and silicon substrates, respectively. Indeed, these nonconventional methods of polymorph screening have the potential to reproducibly generate nonequilibrium polymorphs by an efficient control of process parameters.^28,29^

Functionalized [1]­benzothieno­[3,2-*b*]­[1]­benzothiophene (BTBT) derivatives are ideal candidates because of their high solution processing and chemical stability.^30,31^ Numerous research groups have reported the study of BTBT derivatives with flexible alkyl side chains^25,32–37^ or bulky groups.^38–41^ The alkyl chains favor the presence of liquid crystal phases^31,42–45^ due to conformational flexibility, while the *tert*-butyl group favors the order–disorder transition.^46^ In our study, we aim to investigate the structural changes that occur when flexible alkyl chains and bulky groups are combined within the same molecule. We thus selected a BTBT derivative containing C6 alkyl chains ending with a *tert*-butyl group 2,7-bis­(7,7-dimethyloctyl)­benzo­[*b*]­benzo­[4,5]­thieno­[2,3-*d*]­thiophene (ditBuC6-BTBT) (Figure 1).

**Figure 1:**
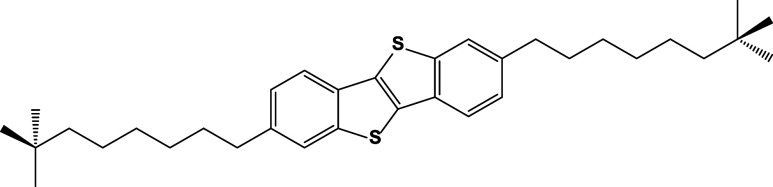
Chemical structure of ditbuc6-BTBT.

Herein, we report wide, bulk, and thin-film polymorph screening and the study of thermodynamic stability and structural properties of the observed polymorphs. Conventional methods of recrystallization led to the finding of three polymorphs existing at room temperature (RT), named Forms I, Ia, and II, while another polymorph (Form III) is observed only at high temperatures, i.e., above 100 °C. Both Form I and Form Ia can be described as ‘disappearing polymorphs’ because it became very difficult to crystallize both of them after the discovery of the thermodynamically stable polymorph (Form II). Forms I, Ia, and II exhibit rigid molecular packing, indicating limited conformational disorder of the alkyl chains, consistent with the behavior typically observed in low-temperature stable crystalline polymorphs of alkylated BTBT derivatives. To attain more control over crystallization conditions and to investigate more polymorphs, we explored nonequilibrium processing techniques leading to the production of thin films, namely, the thermal gradient and solution shearing methods. We further calculated transfer integrals and evaluated the ionization energies (IE) of polymorphs Ia and II that show quite different crystal packing. Transfer integrals between highest occupied molecular orbital (HOMO) [lowest unoccupied molecular orbital, LUMO] levels of adjacent molecules illustrate the ease of exchanging a hole [electron] between two molecules; large values thus favor charge delocalization. The IE represents the energy required to extract an electron from a system, providing insight into electron delocalization as well as electrostatic and polarization effects within the crystal packing. According to Koopmans’ theorem, the first ionization energy of an isolated molecular system corresponds to the negative of the HOMO energy, assuming that the orbitals of the neutral molecule remain unchanged upon ionization.^47^ In the solid state, the IE of an OSC material is not a single value, as it is for an isolated molecule; instead, multiple values emerge due to the anisotropy of ordered molecular assemblies, similar to the variations observed across different crystallographic orientations in metals.^48^ Ideally, the best-performing OSCs in organic field-effect transistors (OFETs) have IEs ranging from 5.1 to 5.3 eV, which facilitates efficient charge injection from high-work-function electrodes while ensuring stability against oxidation.^25,49^ An effective OSC candidate should also exhibit low reorganization energy along with large and directionally balanced transfer integrals. In herringbone packing, it is particularly beneficial if the transfer integrals along different directions share the same sign, as this constructive overlap supports efficient hole transport.^50^

Later on, we fabricated OFETs using solution shearing and thermal evaporation. In our case, the observed deep IE value is reflected in the electrical performances of the OFETs.

## Experimental Section

### Synthesis

DitBuC6-BTBT was synthesized according to the procedure detailed in the Supporting Information and illustrated in Scheme S1. All solvents and reagents, including those obtained from Sigma-Aldrich and other commercial suppliers, were used as received without further purification.

### Polymorph Screening

Solubility screening was performed for ditBuC6-BTBT using 21 different solvents prior to further study (Table S1). Solubility was examined at room temperature (RT), 50, and 75 °C (depending on the solubility and the respective boiling point of solvents).

Recrystallization of ditBuC6-BTBT was carried out by solvent evaporation at RT in chloroform (CHF), dichloromethane (DCM), anisole (ANI), 1,2-dimethoxyethane (DMX), isopropyl acetate (IPA), isopropyl ether (IPE), methyl ethyl ketone (MEK), *p*-xylene (PXY), tetrahydrofuran (THF), and toluene (TOL); at 50 °C in diethyl carbonate (DEC), *N*,*N*-dimethylacetamide (DMA), *N*,*N*-dimethylformamide (DMF), and 1-methyl-2-pyrrolidone (MPY); and at 75 °C in 2-propanol (2PR), benzyl alcohol (ABZ), and ethanol (ETH) (Table S2). Recrystallization by antisolvent addition was carried out by adding the antisolvent at RT to a saturated solution: the solvent systems used are acetonitrile: *p*-xylene (1:1 v/v), chloroform: 2-propanol (1:1 v/v), chloroform: dimethyl sulfoxide (DMS) (1:1 v/v), tetrahydrofuran: ethanol (1:1 v/v), and toluene: ethanol (1:1 v/v). Prolonged slurry maturation experiments at RT were performed by stirring Form I in 2-methoxyethanol (2MX), acetonitrile (ACN), DMA, DMF, DMS, ETH, and H_2_O. Slurry with solvent mixtures was also performed. Controlled recrystallization by a temperature gradient has been performed using the platform Crystal16 by Technobis, cooling a clear solution (5 mg mL^–1^) in DMA, DMF, ETH, and TOL at a constant rate of 0.125 °C/min from 70 °C to RT. The starting material was also tested with mechanochemistry by dry grinding (Table S2).

### Single-Crystal X-ray Diffraction (SCXRD)

Crystals of Form Ia and Form II of ditBuC6-BTBT for single-crystal X-ray diffraction were obtained from CHF (1 mg mL^–1^) by solution evaporation and DMA solutions (1 mg mL^–1^) by temperature gradient crystallization in Crystal16, respectively. Crystallographic parameters of all of the polymorphs are reported in Table 1.

**Table 1:** Crystallographic Parameters of All Polymorphs

parameters	form I	form Ia	form II	form III
formula	C_68_H_96_S_4_			
molecular weight (g mol^–1^)	520.84			
temperature (K)	293(2)	293(2)	293(2)	411(2)
crystal system	monoclinic	monoclinic	triclinic	triclinic
space group	*C*2	*Cc*	*P*1̅	*P*1̅
*a* (Å)	14.317 (8)	11.849(3)	5.903(10)	8.034 (6)
*b* (Å)	18.119 (6)	11.839(11)	19.606(2)	14.297 (6)
*c* (Å)	6.224 (2)	42.823(7)	27.750(3)	20.621 (1)
α (°)	90	90	105.525(10)	103.248 (3)
β (°)	107.904 (5)	90.752(15)	94.938(12)	77.402 (5)
γ (°)	90	90	98.078(11)	87.129 (3)
*V* (Å^3^)	1536.4 (10)	6006.7(19)	3037.7(7)	2237.4 (2)
*Z*/*Z*′	2/0.5	8/2	4/2	3/1.5
density (g·cm^–3^)		1.152	1.139	
*F* (000)		2272	1136	
μ (mm^–1^)		0.198	0.196	
GOF on *F* ^2^		1.074	1.032	
*R*_1_ on *F*, *I* > 2σ(*I*)/*R*_ex_		0.1132	0.1133	
W*R*_2_ (*F* ^2^ all data) *R*_wp_		0.2959	0.1642	
*R*_wp_ (Pawley)	2.97			1.84
CCDC number		2360690	2360689	

All the crystal structures were collected with a Rigaku-Oxford Diffraction Xcalibur S diffractometer equipped with a Mo Kα (λ = 0.71073 Å) X-ray source, a graphite monochromator, and a Cryostream 800 cooler. Both Form Ia (CCDC 2360690) and II (CCDC 2360689) were collected at 293 K. Both crystal structures were solved using the OLEX2 1.5 software-SHELXT codes and refined with SHELXL (version 2018/3). For better visualization, crystal structures were digitalized on CCDC Mercury 2020.3.0.^51,52^ Due to the poor crystal quality, the data collection of Form Ia was not ideal, and the data for Form Ia were solved and refined by keeping all atoms isotropic to avoid encountering nonpositive definite results. Crystals of Form II exhibited a thin, needle-like morphology and demonstrated flexibility, making them susceptible to damage during handling and data collection (see the Supporting Information).

### Powder X-ray Diffraction (PXRD)

Qualitative PXRD to identify the crystalline form was collected with a Rigaku MiniFlex 600 diffractometer with Cu Kα radiation from a copper-sealed tube operated at 40 kV voltage and 15 mA current using a Bragg–Brentano geometry. Diffraction patterns were measured over the 2θ range of 2–40° by step scanning with an increment of 0.01° per step.

### Thin-Film X-ray Diffraction

The XRD data of ditBuC6-BTBT films were also collected with a Rigaku MiniFlex 600 diffractometer with Cu Kα radiation at room temperature.

The films prepared by directional crystallization were collected on a Panalytical diffractometer with two-dimensional (2D) area detectors PIXcel3D using parallel beam geometry, in the 2θ range between 2 and 40° with an increment of 0.02° per step.

The XRD ditBuC6-BTBT devices prepared by the bar-aided meniscus shearing (BAMS) technique were collected on a D-5000 model Siemens diffractometer with a secondary monochromator and scintillation detector.

Thin films obtained by evaporation were characterized by X-ray diffraction (Rigaku SmartLab) performed on 40 nm thick films deposited through vacuum deposition onto TDPA/Al_2_O_3_ substrates held at 25, 40, 70, 100, and 130 °C. The diffractometer is equipped with a Cu Kα source. The measurements were carried out at a tube voltage of 40 kV (tube current of 50 mA), with scanning steps of 0.04° and a scanning speed of 1.5°/min.

### Thermogravimetric Analysis-Evolved Gas Analysis (TGA-EGA)

TGA-EGA analysis was performed to determine the thermal stability and to obtain information about the purity of the ditBuC6-BTBT powder. The measurement was performed on approximately 6 mg of the sample on a Mettler-Toledo TGA coupled with a Thermo Nicolet iS 10IR FT-IR spectrometer operated at a scan rate of 10 °C min^–1^, and the spectra were processed using STARe software.

### Differential Scanning Calorimetry (DSC)

The DSC analysis for all of the samples was performed on a Mettler-Toledo DSC1 instrument. Approximately 2–4 mg of samples were crimped in hermetic aluminum crucibles (40 μL) and scanned from room temperature to 200 °C at a heating rate of 2, 5, and 10 °C min^–1^ under a dry N_2_ atmosphere (flow rate 80 mL min^–1^). The data were treated with the STARe software.

The ultrafast DSC measurement was recorded using a PerkinElmer Diamond differential scanning calorimeter. Both samples of Forms I and II were placed in open Al-pans. All measurements were conducted in the 20–220 °C temperature range at a scan rate of 300 °C min^–1^.

### In Situ Variable-Temperature X-ray Diffraction (VTXRD)

VTXRD was performed at the Paul Scherrer Institut (PSI) Synchrotron radiation facility (Switzerland). PXRD in capillary transmission mode at the MS-X04SA beamline from 24 to 148 °C for the starting material (Form I) of ditBuC6-BTBT. The beam energy of 12.4 keV (1.0 Å) was used for data collection. The MS powder diffractometer is operated in Debye–Scherrer geometry and equipped with a solid-state silicon microstrip detector called MYTHEN (Microstrip sYstem for TimerEsolved experimeNts). Starting from room temperature, the XRD pattern was collected at various intervals until the complete conversion of Form I to Form III (138 °C).^53^

### Microscopy

#### Hot Stage Microscopy (HSM)

Crystals placed on a glass slide and covered with a coverslip were transferred to a heating chamber (hot stage) on an OLYMPUS BX41 stereomicroscope equipped with a LINKAM LTS350 stage for temperature control and VISICAM analyzer. The heating chamber was capped with a sealable lid during heating and cooling cycles, and the rate was kept constant at 10 °C min^–1^. Time-lapse images were taken using a NIKON DS FI3 high-speed camera for all in situ experiments, and the images were analyzed using software Nikon NIS Elements and Linksys32 data capture.

#### Optical Microscopy

Optical microscope pictures for OFETs fabricated with BAMS were taken using an Olympus BX51 equipped with a polarizer and analyzer.

For OFETs fabricated by evaporation, optical microscope images were taken with a Zeiss Axiotron equipped with Zeiss Mikroscope Objektiv Epiplan-Neofluar lenses and an AxioCam MRc.

### Temperature Gradient Apparatus

The setup consists of a Linkam GS350 system, presenting two distinct heating stages separated by a gap. One heating stage was set at a temperature above the melting point (*T*_h_), and the other stage was at a temperature below the crystallization point (*T*_c_) of ditBuC6-BTBT. The distance (gap) between the two stages, where the thermal gradient was generated, was 2 mm. A 76 mm × 26 mm × 1 mm microscope glass slide (Marienfeld Cat. No. 1000000) was intercalated between the stages and the sample to ensure a constant displacement velocity of the sample. During our thermal gradient experiments for ditBuC6-BTBT, the hot stage was set at temperature *T*_h_ = 170 and 180 °C, while the cold stage temperature *T*_c_ was varied from 70 to 140 °C. The system was covered by a hermetic lid so that the system remains thermally independent of the laboratory environment. This setup was mounted on a polarized optical microscope (POM) to take images before, during, and after the experiment.

#### Sample Preparation

We used 20 × 20 × 0.16 mm^3^ D263 Borosilicate cover glasses (Cat. No. 0101040, Marienfeld, Germany). The glass substrates were first washed with toluene and isopropyl alcohol and then dried with a nitrogen gun, followed by UV-ozone treatment for 20 min for all substrates. After this, 3–4 mg of ditBuC6-BTBT sample was deposited on the glass substrate, which was sandwiched and melted in the hot stage of the thermal gradient setup.

FKM treatment on the glass substrates was also performed to observe the influence of the substrate on the nucleation mechanism and, thus, on polymorphism. FKM is a fluorinated rubber [(CH_2_–CF_2_)_0.6_–(CF_2_–CF­(CF_3_))_0.4_]_n_ with molecular weight *M*_w_ = 70,000 g mol^–1^. The FKM solution was prepared in acetone (60 mg mL^–1^) and kept overnight, stirring at 1000 rpm. The solution was then filtered by using a 5 μm phobic filter. This FKM solution was then spin-coated at various amounts of FKM (μL) on the cleaned substrates at a 6000 rpm spin-coating speed with a constant acceleration of 4000 s.

#### Calibration of the Magnitude of the Temperature Gradient Setup (*G*_cal_)

Previous work has shown that the effective magnitude of the temperature gradient (*G*_exp_) that takes place between the hot and cold zones is less than the magnitude calculated by the equation G = (*T*_h_ – *T*_c_)/*x*, where *x* = 2.0 mm (the gap between the hot and cold stages).^42^ For our experiments, the temperature gradient was calculated to be *G*_exp_ ≈ 50 °C mm^–1^ and *G*_exp_ ≈ 15 °C mm^–1^ for the *T*_h_ – *T*_c_ couple 170–70 °C and 170–140 °C, respectively. The cooling rate *C* at the growth front was calculated by the equation *C* = (*T*_h_ – *T*_c_)*V*/*x*, where *V* is the pulling velocity.^42^

### Ionization Energy (IE)

Photoelectron yield spectroscopy (PYS) in air was used to determine IE values from the photoelectron emission yield of OSC samples in the form of powder. Photoelectron yield curves were collected within an energy range of 3.4 to 6.2 eV by using a Riken Keiki spectrophotometer (Japan) model AC-2 with an energy step of 0.05 eV and a UV spot intensity of 100 nW. The final estimate for IE values is known with an experimental error of ±0.05 eV or less.

### Transfer Integrals

Transfer integrals were computed within a fragment approach with the ADF package using the B3LYP functional and a DZ basis set.^54^ We computed them for each pair in a 3 × 3 × 3 supercell extracted from the crystal structure to assess the dimensionality of transport.

### OFETs Fabrication and Characterization

#### OFET Fabrication with Bar-Assisted Meniscus Shearing (BAMS)

##### Bottom Gate/Bottom Contact (BGBC) Devices

200 nm SiO_2_/Si substrates, *C* = 17.26 nF cm^–2^ were used. Interdigitated electrodes, fabricated through photolithography, were composed of Cr (5 nm) and Au (40 nm), deposited via thermal evaporation at deposition rates of 0.1–0.5 and 1–5 Å s^–1^, respectively (Micro-Writer ML3 from Durham Magneto Optics). The channel lengths for BGBC devices were 100 and 150 μm, and the channel width/length ratio was always set to 100. The substrates were sonicated in acetone and isopropanol for 15 min, followed by 25 min of UV-ozone treatment to avoid dewetting during the BAMS deposition. The substrates were then immersed in a 15 mM solution of pentafluorobenzenethiol (PFBT) in isopropanol for 15 min to modify the work function of Au contacts. PFBT was purchased from Sigma-Aldrich. Finally, the substrates were rinsed with pure 2-propanol and dried under a nitrogen flow.

##### Bottom Gate/Top Contact Devices (BGTC)

The silicon substrates were cleaned with acetone and 2-propanol as mentioned above. After the solution shearing deposition, the films were mounted on the stage with shadow masks with a channel width of W = 4000 μm and channel lengths of *L* = 50–200 μm. The stage was carefully placed in the thermal evaporator, and the chamber was kept under vacuum for 3 h using the Leybold screen operator. After 3 h, the Au deposition was started. The program was set for a Au thickness of 25 nm. After evaporation, samples were kept in the dark for 7 days prior to measurement.

##### Organic Semiconductor Solution Deposition

The pristine ink consisted of 2 wt % solutions of ditBuC6-BTBT in chlorobenzene which was dissolved by heating overnight at 105 °C. Polystyrene (PS) (*M*_w_= 10,000 (10k) g mol^–1^) was purchased from Sigma-Aldrich and used without further purification. A blend solution of ditBuC6-BTBT and PS in chlorobenzene 2 wt % was prepared at a ditBuC6-BTBT:PS weight ratio of 4:1. Both pristine and blend films were deposited by the BAMS technique in ambient conditions at 105 °C and a coating speed of 10 and 1 mm s^–1^.

##### Electrical Measurements

Transistor measurements were carried out with an Agilent B1500A semiconductor device analyzer at ambient conditions. For all transfer measurements, the *V*_DS_ was −5 V (linear) and −40 V (saturation). The devices were characterized by extracting the field-effect mobility in linear and saturation regimes and threshold voltage (*V*_th_). The charge mobility μ was extracted using the following equation:

1 $$\mu^{\mathrm{lin}}=\frac{{\partial I}_{\mathrm{DS}}}{{\partial V}_{\mathrm{GS}}}\frac{L}{\mathrm{CW}V_{\mathrm{DS}}}$$

2 $$\mu^{\mathrm{sat}}={\left(\frac{\partial \surd I_{\mathrm{DS}}}{\partial V_{\mathrm{GS}}}\right)}^{2}.\frac{2L}{W}.\frac{1}{C}$$

where *I*_DS_ is the source-drain current, *V*_GS_ is the applied source-gate voltage, *L* is the channel length, *W* is the channel width, *C* is the specific capacitance of the dielectric, and *V*_DS_ the applied source-drain voltage.

#### OFETs Fabricated by Evaporation

OFETs were fabricated on the heavily doped silicon wafer (30 nm thick Al_2_O_3_ layer), which served as the gate electrode, and were provided by the manufacturer (Christian-Albrecht University of Kiel, Institute for Electrical Engineering, and Information Technology). The Al_2_O_3_ surface was pretreated with oxygen plasma (Diener Electronic; oxygen flow rate 20 sccm, pressure 0.50 mbar, plasma power 100 W, duration 2 min) and then immersed overnight in a 1.5 mM solution of *n*-tetradecylphosphonic acid (TDPA, Sigma-Aldrich) in 2-propanol (Acros Organics). Afterward, the substrates were rinsed with 2-propanol, dried with N_2_ flux, and put on a hot plate at 100 °C for 10 min. This treatment leads to the formation of a self-assembled monolayer (SAM) of TDPA on the Al_2_O_3_ surface^55,56^ resulting in an additional dielectric thickness of ca. 1.5 nm. The TDPA/Al_2_O_3_ dielectric has a calculated capacitance of 185 nF cm^–2^. Gold source and drain contacts were thermally deposited through a shadow mask onto the substrates held in a vacuum at room temperature (UNIVEX 300, Leybold GmbH; pressure of ∼10^–5^ mbar, deposition rate of 0.7 Å s^–1^, nominal thickness of ca. 50 nm). Next, a SAM of PFBT (Alfa Aesar) was obtained by immersing the substrates in a 10 mM solution of PFBT in 2-propanol for 30 min, then rinsed with 2-propanol, and dried with an N_2_ flux. The OSCs were deposited through a shadow mask onto the substrates (held at 25, 40, 70, 100, and 130 °C) by thermal evaporation in a vacuum (UNIVEX 300, Leybold GmbH; the pressure of ≈ 9 × 10^–7^/3 × 10^–6^ mbar, the deposition rate of 0.3 Å s^–1^, the nominal thickness of ca. 25 or 40 nm). The nominal thickness of OSC films and gold electrodes was monitored via crystal quartz microbalance and confirmed by ellipsometry (OSC films) and profilometry (gold electrodes). The obtained OFETs present a channel width of 480 μm and a channel length of 215 μm. The electrical measurements were performed in ambient air and room temperature (Agilent 4155C Semiconductor Parameter Analyzer). Mobility values in linear (μ_lin_) and saturation regime (μ_sat_) were calculated using the gradual channel approximation model as expressed in eqs 1 and 2.

## Results and Discussion

### Polymorph Screening

Following the synthesis of ditBuC6-BTBT as reported in the Supporting Information and Scheme S1, the compound’s purity was confirmed by ^1^H NMR, 13C-NMR, high-resolution mass spectrometry (HRMS) spectra, and HPLC analyses (Figures S1–S9). After this, the solubility assessment of ditBuC6-BTBT was performed, which reveals that it is soluble in most of the organic solvents that were tested (Table S1), and hence, it was recrystallized in various solvents (Table S2). The explored methods were evaporation, antisolvent addition, slurry maturation, recrystallization by temperature gradient, and mechanochemistry (Table S2).

The starting material is a crystalline powder and was referred to as Form I. We observed two new polymorphs for ditBuC6-BTBT at room temperature by polymorph screening recrystallization experiments. Solvents like ANI, CHF, DCM, IPE, PXY, THF, and TOL yielded Form Ia with a flat-plate-like morphology. Thin hair-like long needle crystals of Form II resulted from controlled recrystallization in Crystal 16 (a multireactor crystallizer by Technobis) by the temperature gradient in DEC and DMX solvents. In some solvents like DMA, DMF, IPA, etc., a mixture of Form Ia and Form II was obtained as concomitant polymorphs, but after some days (days varied for each different solvent), the mixture eventually converted to Form II. The stability assessment of both forms by slurry experiments suggests that Form II is the thermodynamically stable polymorph at room temperature. The pristine Form I was obtained only from the first crystallization performed during the synthesis of the molecule.

### Thermal Properties and Phase Transitions

Thermal gravimetric analysis results indicate that ditBuC6-BTBT is stable up to approximately 400 °C (Figure S10), whereas DSC curves on the starting material (Form I) at 5 °C min^–1^ showed only the melting onset at 145 °C. DSC of Form II at 5 °C min^–1^ shows an endothermic peak at 97 °C, indicating a solid–solid phase transition to a high-temperature polymorph Form III and then melting at around 145 °C (Figure S11). It is noteworthy that the melting temperatures observed in the two DSC curves were identical, indicating that Form I is likely to undergo a phase transformation into Form III too. To verify temperature-dependent phase transitions, we also carried out hot stage microscopy (HSM) and variable-temperature X-ray diffraction (VTXRD) at the PSI synchrotron.

The HSM shows a transition in crystals of Form Ia→Form III that occurs in a very long temperature range, where the phase transition appears to swipe across the crystal from one end to another, which is visible from the POM images (Figures S14 and S15). In the phase transition from Form II→Form III, the needle-like crystals of Form II appear like “tail-wagging” as the crystal merely moves or changes the face orientation from one end of the needle (Figure S16).

In the VTXRD measurement of Form I ditBuC6-BTBT, we observed a phase transition onset from 80 °C, which occurred until 138 °C and the high-temperature phase is ascribable to Form III (Figures 2 and S17). This conversion observed in VTXRD explains the similar melting temperatures observed in DSCs, as both Form I and Form II undergo the transition to Form III. One possible explanation for the fact that no transition peak was observed in DSC of Form I could be that the transition is extended in a very wide range of temperatures so that the peak almost appeared flat. Indeed, by the DSC at an ultrafast rate (300 °C min^–1^), we could capture the transition Form I → Form III, which started at 60 °C followed by the melting (Figure S12). We also carried out the DSC measurement at 300 °C min^–1^ for Form II, and the same transition of Form II → Form III was observed starting at 110 °C followed by melting (Figure S13).

**Figure 2:**
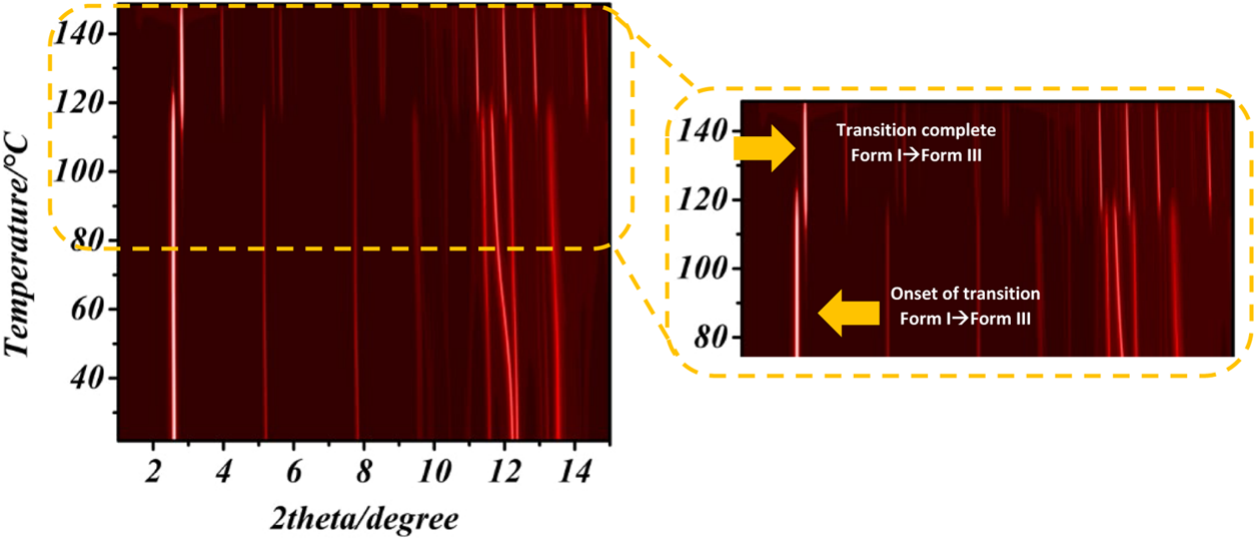
VTXRD (λ = 0.9999613 Å) showing the transition of Form I to Form III.

Upon cooling from the high-temperature Form III, it initially transforms into Form I, which gradually converts into Form II over the course of a few days.

## Structural Characterization by Single-Crystal X-ray Diffraction

We selected suitable single crystals of both Form Ia and Form II for the SCXRD measurement. All the PXRD pattern comparisons of different polymorphs are presented in Figure S18. The crystal quality of Form Ia was not ideal, and it was difficult to isolate a suitable single crystal for the SCXRD measurement. Form Ia was solved in the monoclinic space group *Cc* with two full molecules in the asymmetric unit. This polymorph packs with 8 molecules in the unit cell (*Z*′ = 2), comprising two antiparallel layers. Both the molecules in the asymmetric unit are superimposable with a root-mean-square deviation (RMSD, defined as the square root of the mean squared error) of 0.063. The layers that are parallel to the plane (0 0 1) present a quite smooth surface. Inside the layers, the molecules are arranged in a herringbone packing motif with an angle of 59.89° (Figure 3a) and a tilt angle of 40° with respect to the (0 0 1) plane (Figure 3b). Previously, it was observed that the core-tilt angle decreases with chain substitutions. For example, bare BTBT has almost an upright core with a tilt angle of 87°^38^ and the symmetrical substitution of BTBT core with the long alkyl chains like C6-diol (83.99°),^34^ C7 (87.66°),^43^ C8 (87.87°),^57^ C12 (87.90°),^32^ etc. does not significantly modify the tilt angle; in contrast, the introduction of bulky groups as side chains- like diiPr (67°), ditBu (50°) or diTMS (41°), significantly decreases the tilt angle which diminishes orbital overlap between neighboring molecules and the charge transport properties.^39^ Since, in our case, we have the combination of alkyl-C6 and bulky-tBu chains, the tilt angle is expected to be decreased.^58^

**Figure 3:**
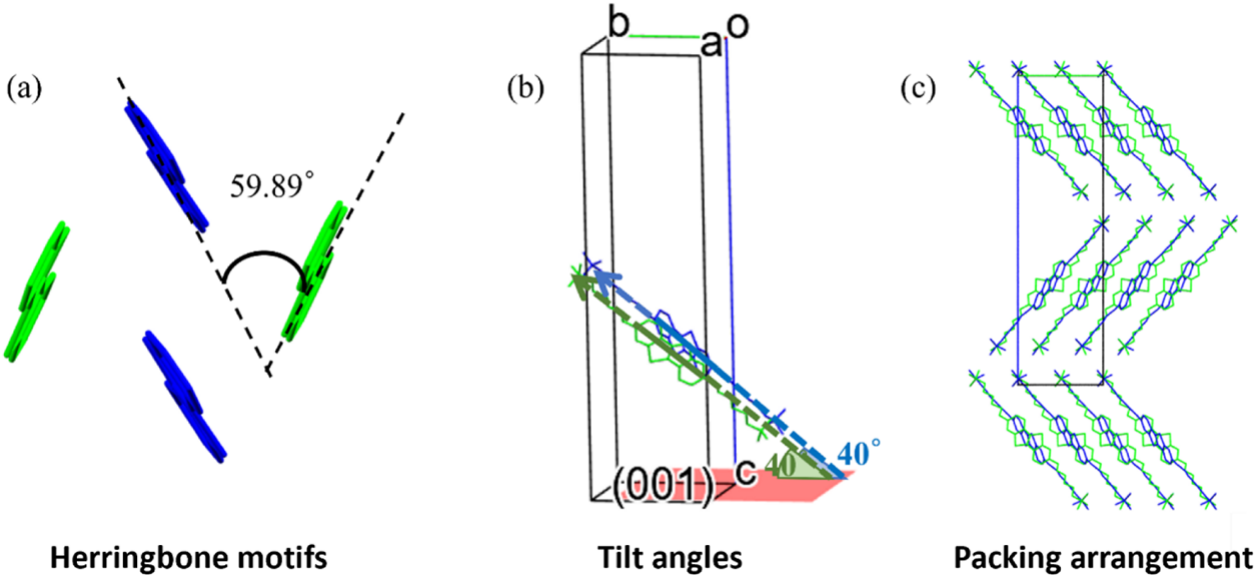
Crystal structure of Form Ia, showing (a) a herringbone motif with an angle of 59.89°, (b) a core-tilt angle (40°) of both molecules in the asymmetric unit, and (c) a molecular packing arrangement. In (a), hydrogens and alkyl chains are removed for clarity; in (b) and (c), only hydrogens are omitted. The two crystallographically independent molecules are shown in blue and green.

Form II crystals were less tricky to isolate as compared to Form Ia; however, the crystals were extremely flexible and thin needle-shaped, which led to difficulty in crystal mounting and data collection. Some good quality and collectible crystals were found in the recrystallization experiment from DMA solvent, and the data collection was performed at room temperature. Form II was found to crystallize in a triclinic system (*P*1̅), with one full molecule and two half-molecules placed on the inversion center in the asymmetric unit (*Z*′ = 2). Two of the independent molecules have almost the same tilt angle with respect to the (0 0 1) plane (54 and 56°), while the third molecule adopts a tilt angle of 42° (Figure 4).

**Figure 4:**
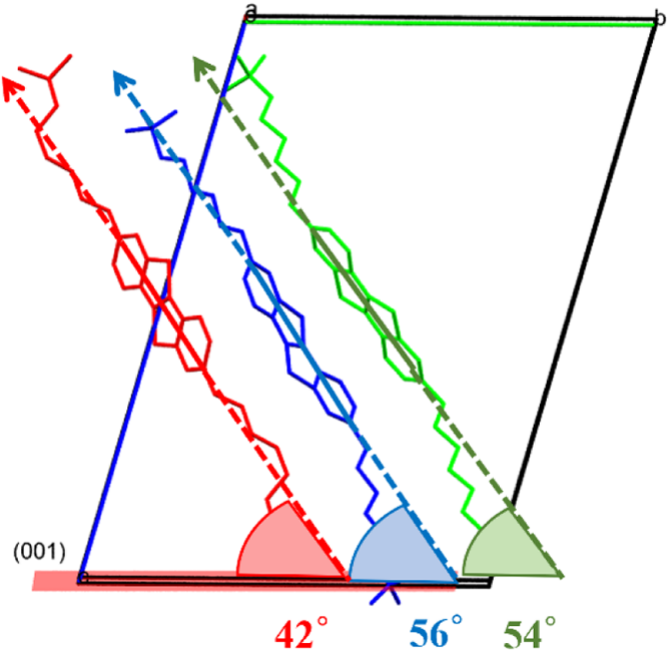
Three independent molecules in the unit cell of Form II (colored according to symmetry equivalence) and their corresponding tilt angles. The asymmetric unit consists of a half-red molecule, a half-green molecule, and a full-blue molecule. The blue and green molecules exhibit similar tilt angles of 56 and 54°, respectively, while the red molecule shows a reduced tilt of 42° relative to the (001) plane.

The third molecule (indicated in red color in Figure 4) is also shifted with respect to the other, and it is not possible to describe it as a herringbone (Figure 5a). The core of the fully independent molecule which does not lie on the inversion center is slightly bent, which could be related to the influence of the alkyl chain. Unlike Form Ia, molecules in the asymmetric unit of Form II are not highly superimposable (RMSD_Red‑Blue_ = 1.026, RMSD_Red‑Green_ = 0.742, and RMSD_Green‑Blue_ = 1.013) and the molecular layers are rough and not distinctly separated (see Figure 5c).

**Figure 5:**
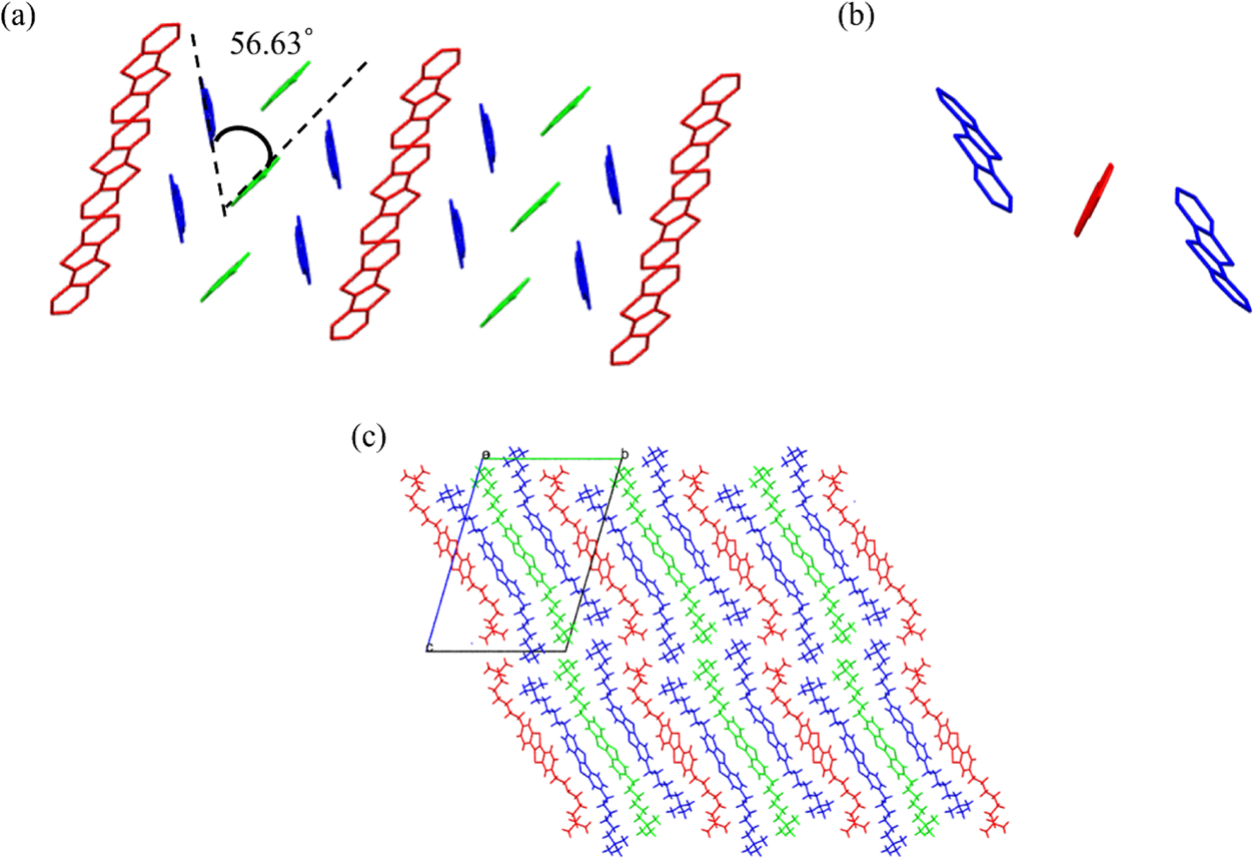
Crystal structure of Form II, showing (a) a herringbone-like motif between green and blue molecules with an angle of 56.63°, (b) absence of a herringbone interaction between red and blue molecules (note the bent-core of the blue molecule), and (c) overall molecular packing arrangement. Hydrogens and alkyl chains are removed in (a), and only hydrogens are removed in (b). The three independent molecules are shown in red, blue, and green.

To further understand the molecular packing and stability of polymorphs Ia and II, we performed pairwise interaction energy calculations using Mercury software.^59,60^ In Form Ia, the molecules adopt a well-ordered herringbone arrangement. These layers are stabilized by strong and consistent intermolecular interactions, with calculated interaction energies ranging from −59 to −74 kJ/mol. This regularity is indicative of balanced intermolecular interactions that give rise to a smooth, densely packed structure (Figure S20). In contrast, Form II displays a more complex packing comprising three crystallographically distinct molecules with varying orientations and tilt angles. The calculated major interaction energies ranged from −58 to −76 kJ/mol. Along with the strong localized contacts (solid red lines, Figure S21b), more networks of weaker peripheral interactions were also reflected in the calculation (blue dotted lines, Figure S20b). Notably, the deviation of one molecule from the herringbone motif introduces geometric and energetic variability, resulting in a more corrugated packing pattern (see also Figure 5c).

Form III is only obtained by solid–solid phase transition of Form I or Form II at a temperature higher than 138 °C so that no single-crystal data could be collected. The powder XRD pattern obtained at high temperature from the PSI synchrotron shows sharp diffraction peaks (in agreement with a crystalline nature), was indexed using TOPAS, and was found to have a triclinic crystal structure and a volume that corresponds to the presence of three molecules in a cell. We were able to get a low *R*_wp_ of 1.84 with Pawley refinement using these unit cell parameters (Figure S22). The strong peak at a low angle (2.80°, λ = 0.9999613 Å) and hence the interplanar distance of 20.39 Å could be attributed to the presence of layers with a lower core-tilt angle promoted by the presence of the bulky group. Despite numerous attempts, it was not possible to solve the structure of Form III, due to the high number of degrees of freedom and/or the presence of disorder at elevated temperatures.

### Form I vs Form Ia

We encountered challenges in distinguishing between the two distinct polymorphs, Form I and Form Ia because they both possess a strong peak 0 0 1 at the same low angle, and the PXRD pattern of both suffers from strong preferential orientation. Due to the similar cell parameters and the preferential orientation of the recrystallized powder and starting material, the observed main peaks are in the same positions making them indistinguishable between Form I and Form Ia. More accurate comparisons were done based on the calculated pattern based on the structure of Form Ia which first was collected at LT (Supporting Information). The mismatch (Figure S23) between the observed and calculated peaks was initially attributed to thermal expansion. Upon collection of the crystal data of Form Ia at RT (which ended up being of a better quality than the data at LT), the mismatch of peaks between the calculated pattern and the high-quality powder diffraction data at RT obtained from the PSI synchrotron remained (Figure 6). We were not able to refine the cell of Form Ia against the powder of the pristine Form I. We then indexed Form I from the PXRD pattern using TOPAS, which resulted in a monoclinic crystal system with a probably *C*2 space group with half a molecule in an asymmetric unit. The Pawley refinement led to a low *R*_wp_ of 2.97 (Figure S25).

**Figure 6:**
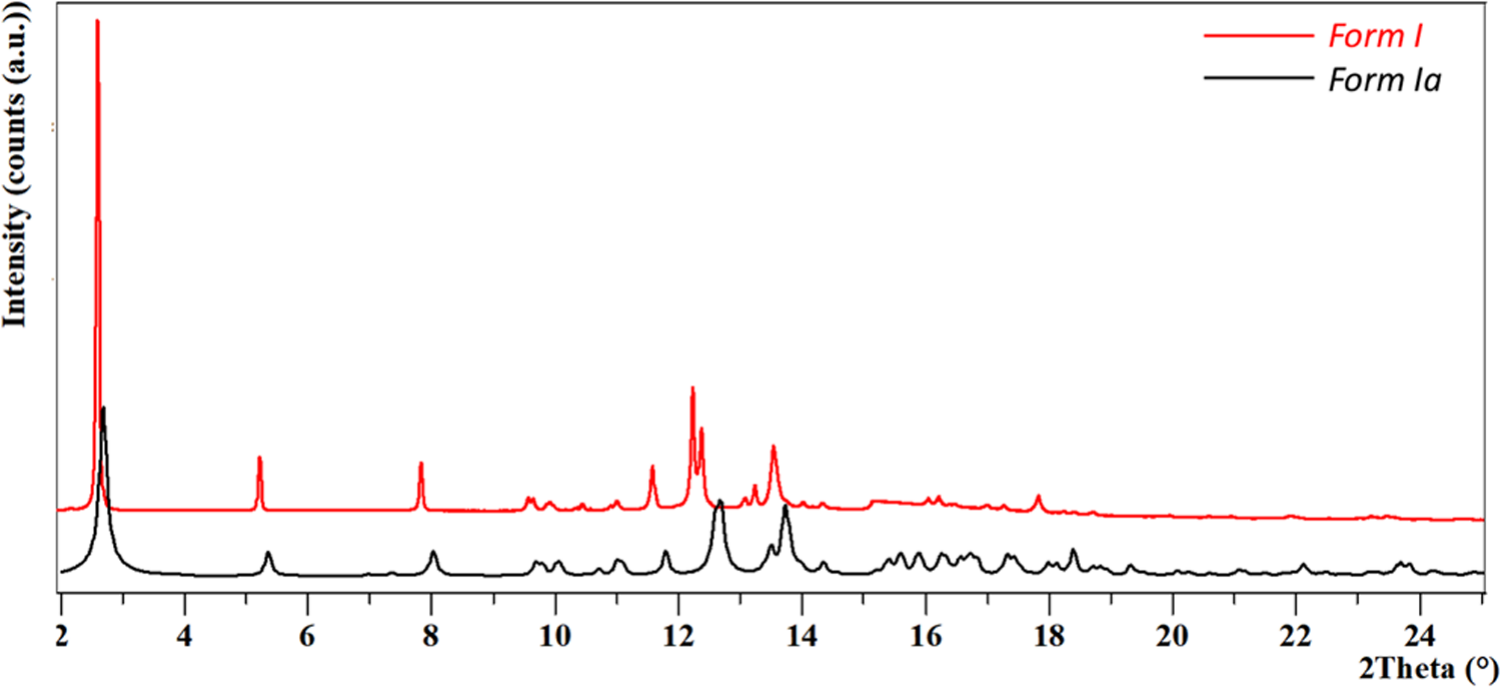
PXRD patterns (RT) of experimental Form I (red) and simulated Form Ia (blue) (λ = 0.9999613 Å) in square root scale.

We also carried out a VTXRD experiment on the starting material (Form I) starting from RT down to 100 K. From the VTXRD_RT_ → _LT_, we did not observe any thermotropic phase change or thermal contraction that could have been the reason for the mismatch of the XRD peaks (Figure S24a), which means that there are two very similar polymorphs- starting material powder (Form I) and the crystals that were recrystallized from solvents (Form Ia) (Figure S24b).

### Are Form I and Form Ia Disappearing Polymorphs?

Based on the polymorph screening performed, it appears that Form Ia often exhibits flat-sheet-like crystals. Form Ia is a kinetic form that is evident from the slurry and solubility assessments. Yet, during the first recrystallization experiments, where we observed only Form Ia, the crystals were found to be stable at RT and could be kept for several months. However, we witnessed a dramatic decrease in the stability of Form Ia when we performed more crystallization experiments for polymorph screening after the discovery of Form II. The presence of seeds of Form II in the surroundings led to unintentional seeding, which triggered the conversion of Form Ia→Form II in a matter of days. Upon conversion, the morphology also changed from sheets to needles. Following a year of polymorph screening and the emergence of Form II, Form Ia became so disfavored that even the recrystallization experiments, which previously produced Form Ia (with sheet morphology), now yield Form II (needle-shaped crystals). Additionally, Form Ia crystals are no longer observed from evaporation. A similar peculiar behavior of vanishing polymorphs was witnessed and reported by Dunitz and Bernstein,^8^ Woodward and McCrone,^61^ and Webb and Anderson,^62^ where the metastable polymorph observed for a certain period was completely displaced by the stable polymorph due to the unintentional seeding. In our case, we found that the conversion of kinetic form to thermodynamic form was occurring only during the recrystallization or in the presence of residual solvent, while there was no such transformation in the thin films or the vacuum-dried crystals. The thin-film XRD of Form Ia was found to be stable for more than a year. However, in the latest fast recrystallizations at low temperature to obtain Form Ia, we always observed concomitant polymorphs with an increasing amount of Form II.

It is worth noting that when it was possible to ascertain the presence of Form I vs Form Ia, we always observed Form Ia except for the starting material; hence, also Form I can be counted as a disappearing polymorph.

## Directional Crystallization Using Temperature Gradient

Bulk polymorph screening with conventional methods often suffers poor process control, leading to elusive polymorphs. Therefore, we explored nonconventional crystallization, where the crystallization parameters can be controlled and both nucleation and growth mechanisms are followed.^63^ With directional crystallization, we produced films from the melt by tailoring the conditions responsible for nucleation and growth, such as the temperature of hot and cold stages, the pulling rate of the substrate, and the cooling rate.

As shown in Figure 7, the nucleation of ditBuC6-BTBT occurs at the edge, or the corner of the substrate (event 1 Figure 7), and the nucleus grows quickly toward the vertical edge in the undercooled region, as the growth follows the coldest slice available and then propagates in the gradient direction (event 2 Figure 7),.^64^ This directional crystallization mechanism enables controlled growth along the temperature gradient, resulting in long oriented crystals suitable for studying polymorphism in thin films. For samples with higher pulling velocities like 50 μm s^–1^, multiple nucleation sites were observed. We explored different conditions by varying the cold stage temperature (*T*_c_ = 70–140 °C), hot stage temperature (*T*_h_ = 170–180°C), the pulling rate of the sample stage (5–50 μm s^–1^), and the cooling rate at the growth front (Table S3). In all cases, we observed the needle-like morphology of the crystals, which aligned uniaxially in the direction of the thermal gradient (event 3 in Figure 7), until the formation of mm^2^ size domains (event 4 in Figure 7). The applied thermal gradient influenced the crystal morphology by promoting anisotropic, unidirectional growth and enhancing the alignment of the crystals across the film (Figure S26).

**Figure 7:**
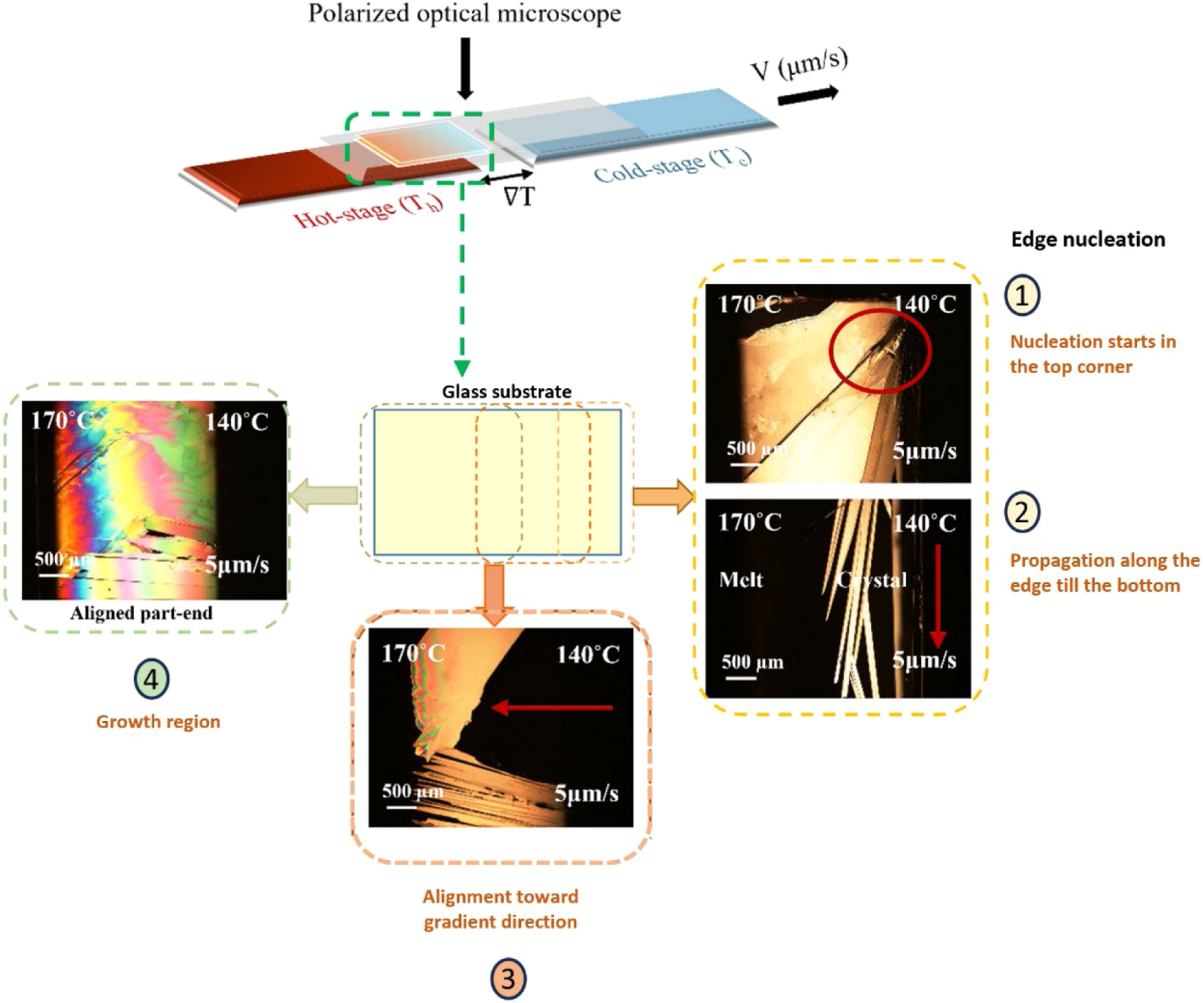
Polarized optical microscopy images during crystallization using the thermal gradient technique. (1) (Right) Nucleation starts in the top corner as the melt reaches the cold stage (*T*_c_), propagating along the edge. (2) Crystals grow parallel to the edge before aligning toward the thermal gradient. (3) Crystals align and compete to form a stable growth front. (4) In the final stage, the growth front has been formed, and only growth and competition between neighboring aligned domains can be observed. Sample conditions: *T*_h_ = 170 °C, *T*_c_ = 140 °C, and pulling velocity = 5 μm s^–1^. The schematic illustrates the sequence of events on the substrate. All the images were recorded at room temperature except for the alignment part to show the crystal growth front.

We also investigated the influence of the surface by treating the glass substrate with fluorinated rubber (FKM).^65^ The polymer was dissolved in acetone and spin-coated onto the glass substrate before sample deposition. Aiming to induce different nucleation conditions, the FKM polymer layer was introduced between the ditBuC6-BTBT film and the glass substrate (Figure S27).

The XRD data of all of the films indicate that when the crystallization occurred, predominantly Form III was observed. However, due to the instability of Form III at room temperature, it starts to transform to Form Ia immediately as the sample is cooled at room temperature. This is evident from the small peak of Form Ia that is present in almost all the samples. This transformation and decreasing stability of Form III were monitored for a month, which illustrated that the kinetics of transformation was very slow. In some of the samples, where the low pulling rate was applied, we could also observe a very small peak of Form II (Figure S28).

We cannot be sure that we observed Form Ia instead of Form I because the strongest peaks of both polymorphs I and Ia are located at the same 2θ values, and the XRD of the films is highly preferentially oriented. Since we always obtained Form Ia during our recrystallizations, we assumed that Form Ia was also obtained in the films.

## Ionization Energy

The ionization energy (IE) of the ditBuC6-BTBT powder and single-crystal samples was determined via photoemission yield spectroscopy (PYS) in ambient air (Table S4). Single crystals provide an ideal platform to investigate the differences in molecular packing and electronic coupling within the crystal lattices of the two polymorphs. The IE values of isolated crystals of Form Ia (5.81 eV) and Form II (5.51 eV) significantly differ by 0.3 eV, thus highlighting the strong influence of structural order on the electronic properties of organic semiconductors.^66^ In fact, the more efficient packing observed in Form II leads to greater stabilization of the HOMO (as confirmed by the significatively larger transfer integrals in the π-stacking direction in Figure 8), resulting in a corresponding decrease in the IE. The IEs of these bulky-end-capped-BTBT polymorphs are larger than those typically observed for linear chain (chain length = n) BTBT derivatives, Cn-BTBT-Cn, typically around 5.3 eV for 5 < *n* < 14,^67^ where a more efficient molecular packing is facilitated by the absence of bulky -tBu groups. Still, these former values lie within the same range as those achieved on previously reported bulky-end-capped BTBTs (around 5.7 eV).^39^ Such large IE values, like those reported for the polymorphs in this study, suggest that injections of charges could be relatively difficult in an OFET device, thus strongly affecting its characteristics, such as carrier mobility and threshold voltage (compared with Table 2).^39^

**Figure 8:**
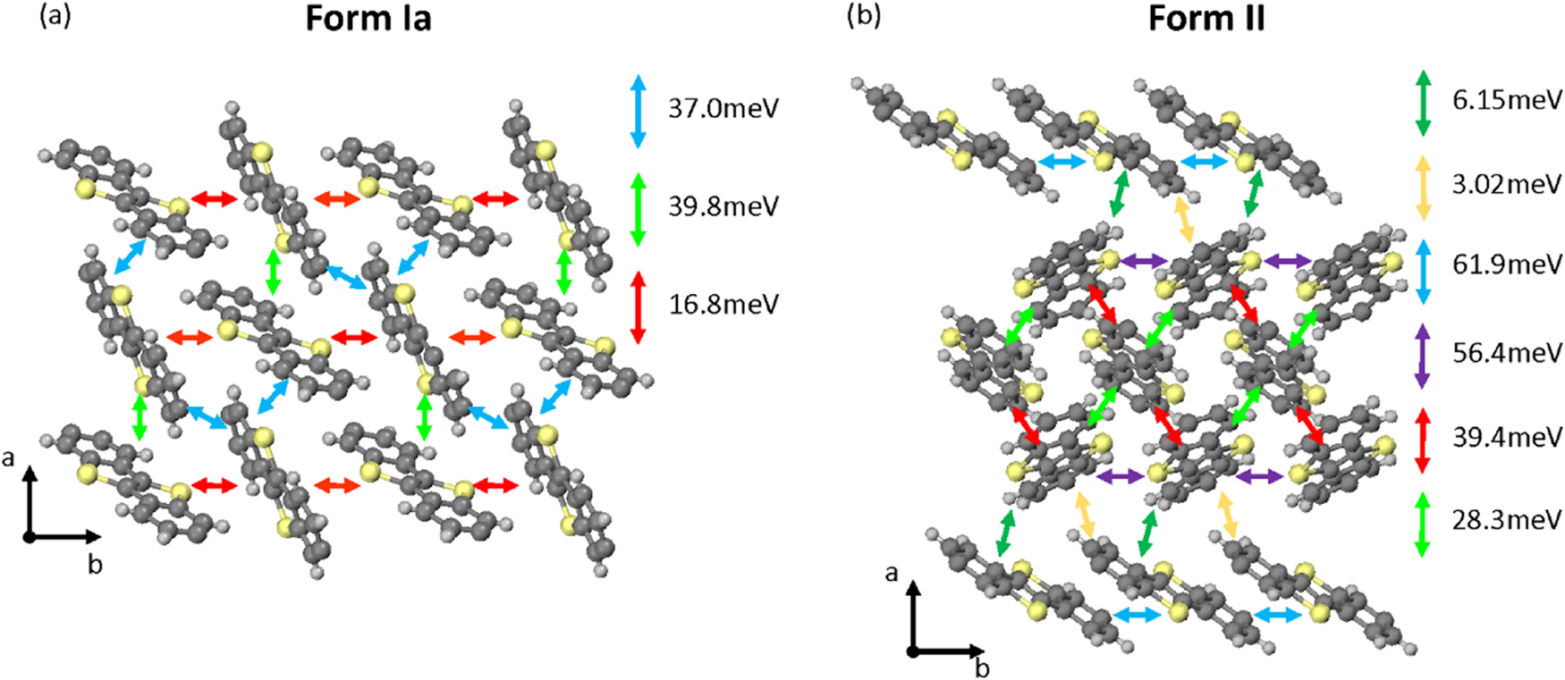
Spatial distribution of transfer integrals of Form Ia (left) and Form II (right).

**Table 2:** Electrical Performances of BC OFETs Based on ditBuC6-BTBT in Linear (*V*_d_ = −0.1 V) and Saturation (*V*_d_ = −4.0 V)^a^

*T*_sub_ (°C)		μ (cm^2^ V^–1^ s^–1^)	*V*_th_ (V)	*I*_ON_/*I*_OFF_
70	linear	0.05 ± 0.01	–3.1 ± 0.1	≈6 × 10^2^
	saturation	0.03 ± 0.01	–3.0 ± 0.1	≈3 × 10^3^

a All of the values are averaged over at least 5 devices.

## Transfer Integrals

The transfer integrals between the highest occupied molecular orbitals (HOMOs) of the individual units were calculated at the density functional theory (DFT) level. Figure 8 illustrates the transfer integrals within the crystal structure of Form Ia and Form II. The Form Ia crystal is made of two shifted herringbone planes that can barely exchange any charge, but the charge transport within these herringbone planes should be rather good, though, as they display transfer integrals of around 40 meV in two directions and a third direction with a significant transfer integral of 16 meV. In Form II, the charge transport is expected within the planes parallel to (0 0 1), but it shows a one-dimensional (1D) transport character with high HOMO couplings along two different stacks, one involving the molecules with a tilt angle of 42̊ which are π-stacked and the second involving the two molecules with 54 and 56° tilt angle within a herringbone motif.

## Thin-Film Polymorph Screening

Films of ditBuC6-BTBT were prepared by solution processing on silicon substrates by varying several parameters like solvents (CHF, TOL, DEC, DMA, and CLB), processing technique (spin-coating, drop-casting, and shear coating (see later)), temperature (pre- and post- thermal treatment up to 110 °C), and concentration (1, 3, and 22.6 mg mL^–1^ (2% w/w)).

The polymorphic tendency of ditbuC6-BTBT in thin films was the same as that in bulk. As observed in bulk, Form Ia was observed in CHF and TOL, while in DEC and DMA, the mixture of Form Ia and Form II was observed, which completely transformed into Form II in time. No other polymorph was obtained from spin-coating or drop-casting experiments.

## Solution Shearing and Device Fabrication

For the fabrication of organic field-effect transistors (OFETs), we exploited the solution shearing deposition technique, as it has appeared to be a promising approach for device fabrication with large area coverage and low production cost.^68^ Moreover, it allows better control of the conditions of crystallization and can induce a polymorph formation different from that found in bulk or in other conventional techniques.

The films of ditbuC6-BTBT were prepared by using the bar-assisted meniscus shearing (BAMS) technique. The OFETs were fabricated using the bottom gate/bottom contact (BGBC) (Figure S29) and bottom gate/top contact approach (BGTC). The use of OSC solutions blended with polystyrene (PS) as a binding polymer was also carried out, as the PS might promote homogeneity and an enhanced thin-film crystallinity and electrical performance of the devices.^68,69^

Hence, we could investigate the impact of shearing deposition on the crystalline property and phase behavior of ditBuC6-BTBT. The POM images were recorded for pristine and PS blended films fabricated at different coating rates and using BGBC and BGTC OFETs configurations (Figure 9). The films deposited with 1 mm s^–1^ shearing rate show poorer coverage, as the active layer was not homogeneous to cover the electrodes. Also, the film morphology was found to be long needles which could be associated with Form III, along with a few plate-like square crystals near the edges as observed in Form Ia.

**Figure 9:**
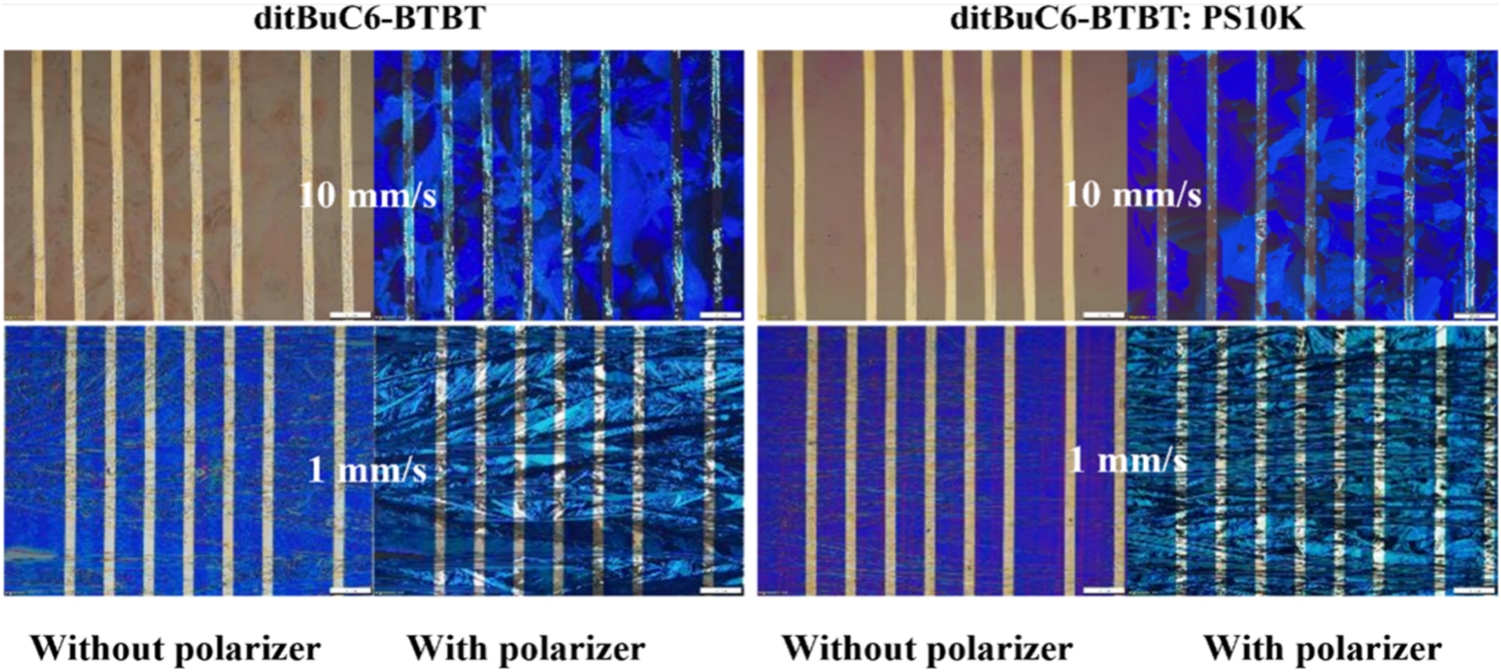
Polarized optical microscopy (POM) images of bottom gate/top contact devices of ditBuC6-BTBT and ditBuC6-BTBT: PS10K prepared with a shearing speed of 10 mm s^–1^ (top) and 1 mm s^–1^ (bottom). Scale bar: 200 μm.

In contrast, the films with 10 mm s^–1^ were more homogeneous and isotropic compared to the films with 1 mm s^–1^, with complete coverage and larger crystalline domains. It was also observed that the film homogeneity was far improved with the PS blend.

From the structural characterization by PXRD of the thin films of all of the samples, we always observed the mixture of Form III and Form Ia, with Form III in dominance (Figure S30). Also, this result agrees with the morphology information obtained from the directional crystallization technique, where we observed needle-like morphology for Form III while Form Ia crystals were sheets or plates.

The presence of two polymorphs in the films by solution shearing makes it even more difficult to isolate a polymorph for electrical measurements or to improve the device performance. Another critical issue associated with the devices of ditBuC6-BTBT is the deep ionization potential values (reported above), which hampers the charge injection between the metal contacts and the OSC, leading to no field-effect response (either in the bottom or top contact configuration).^25^ This was observed in the experimental outcomes of BGBC (Figure S29) and BGTC devices with pristine molecules and ditBuC6-BTBTB: PS ink.

## OFET Fabrication by Evaporation

Due to the inability to obtain operating devices through the BAMS technique, OFETs were fabricated through thermal evaporation in a high vacuum. Typically, ditBuC6-BTBT was deposited onto substrates consisting of a highly doped silicon wafer, which serves as a global gate electrode overgrown by atomic layer deposition with a 30 nm thick layer of Al_2_O_3_. The dielectric was consequently treated with *n*-tetradecylphosphonic acid (TDPA), leading to the formation of a self-assembled monolayer (SAM). During the semiconductor deposition, the substrates were held at temperatures (*T*_sub_) of 25, 40, 70, 100, and 130 °C. Both BGTC and BGBC configurations were fabricated by thermal evaporation of source and drain gold electrodes, resulting in devices with a channel length (*L*) of 215 μm and channel width (*W*) of 480 μm. In the case of BGBC OFETs, the gold contacts were treated with pentafluorobenzenethiol (PFBT), which promotes a more uniform morphology of the organic layer across the contact-active channel interface.^70^

The deposition of ca. 25 nm of ditBuC6-BTBT resulted in nonoperating devices both in the case of BGTC and BGBC geometries at all substrate temperatures. By analyzing the thin films with optical microscopy (Figures S31 and S32), we noted that, for all substrate temperatures, the 25 nm thick layer of ditBuC6-BTBT is not enough to guarantee a uniform coverage of the active channel, giving discontinuous films characterized by pitted morphology. Moreover, thin films deposited at a *T*_sub_ of 130 °C were completely dewetted from the dielectric surface.

Therefore, BGTC and BGBC OFETs were fabricated by depositing a 40 nm thick OSC layer, which results in complete coverage of the active channel at all substrate temperatures (except for *T*_sub_ of 130 °C, which results again in total dewetting of the thin film from the dielectric surface). No drain current modulation upon the application of a gate bias was observed in BGTC devices at all substrate temperatures as well as for BGBC devices with *T*_sub_ values of 25, 40, and 100 °C. BGBC OFETs fabricated with *T*_sub_ of 70 °C were the only ones to show field-effect response, allowing the collection of transfer and output characteristics, depicted in Figure 10. At first, it is clear that ditBuC6-BTBT shows poor performances due to the presence of hysteresis, high threshold voltage (*V*_th_), and low on/off current ratio (*I*_ON_/*I*_OFF_). Particularly, OFETs exhibit *V*_th_ of −3.0 V and I_ON/OFF_ of ≈6 × 10^2^ along with hole mobility (μ) up to 0.05 cm^2^ V^–1^ s^–1^ in the linear regime. The averaged *V*_th_, *I*_ON_/*I*_OFF_, and μ values in the linear and saturation regimes are reported in Table 2. As already mentioned, the main reason for poor electrical performances may be related to the deep ionization potential of ditBuC6-BTBT, which leads to inefficient charge carrier injection, which in turn is reflected in high *V*_th_.

**Figure 10:**
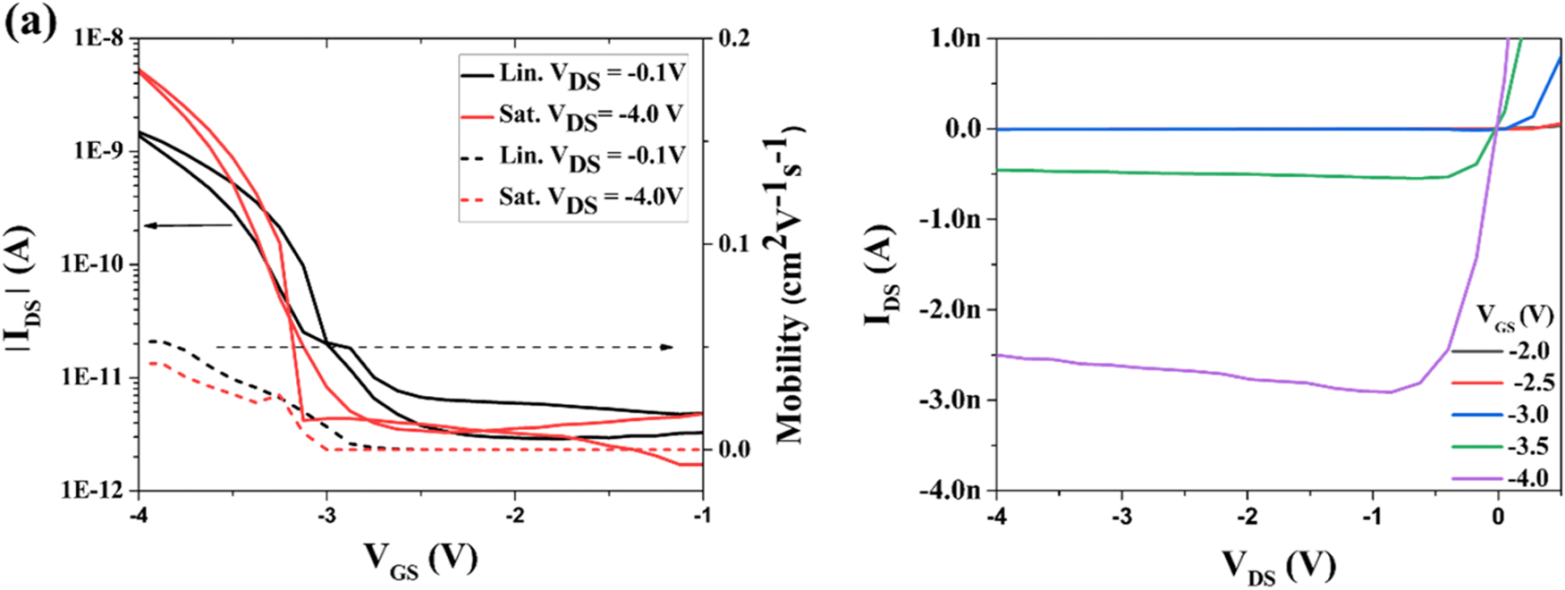
Representative (a) transfer and (b) output characteristics of BGBC OFETs based on ditBuC6-BTBT deposited at *T*_sub_ of 70 °C. In transfer characteristics, solid lines and dashed lines are referred to as drain current and mobility, respectively. OFFTs have W/L = 480/215 μm.

To ascertain the crystal phase and correlate it with the electrical performances of ditBuC6-BTBT-based devices, XRD patterns of the thin films deposited through vacuum evaporation at *T*_sub_ of 25, 40, 70, and 100 °C were recorded (Figure 11). The PXRD data reveals that we obtain a mixture of polymorphs. For instance, at 25, 40, and 70̊ °C, Forms Ia, II, and III are present, with the presence of Form Ia being dominant. Since the PXRD pattern was measured at RT and after some days, we are not sure whether the data report the situation immediately after deposition, or whether the thin film is subject to phase transitions occurring between deposition and PXRD recording. As expected, deposition at 100 °C promotes Form III (same as films produced by solution shearing at 105 °C), which is obtained almost pure, while traces of Form Ia could indicate the beginning of the transition into the stable phase at RT. It is worth noting that the best-performing device was observed for samples at 70 °C, where we can distinctly observe the peaks of three polymorphs. Eventually, the OFETs fabricated by evaporation also resulted in the same problem of obtaining multiple polymorphs in the thin film, as we observed in the solution shearing method. Till now, it remains a challenge to isolate a single polymorph for a device to make any conclusive statement about its respective charge transport properties. The coexistence of three different polymorphs in the OFETs implies the presence of grain boundaries, whose effect on charge transport is certainly detrimental but also renders comparison between polymorph performance impossible. Based on theoretical calculations, Form Ia demonstrates the potential for higher mobility compared to other polymorphs, as indicated by its more favorable set of theoretical charge transport parameters. However, drawing straightforward conclusions is not feasible due to the inability to isolate a single polymorphic form in thin films. This remains a challenge that needs to be overcome to precisely define the structure–property relationships of the polymorphic forms of dtBuC6-BTBT.

**Figure 11:**
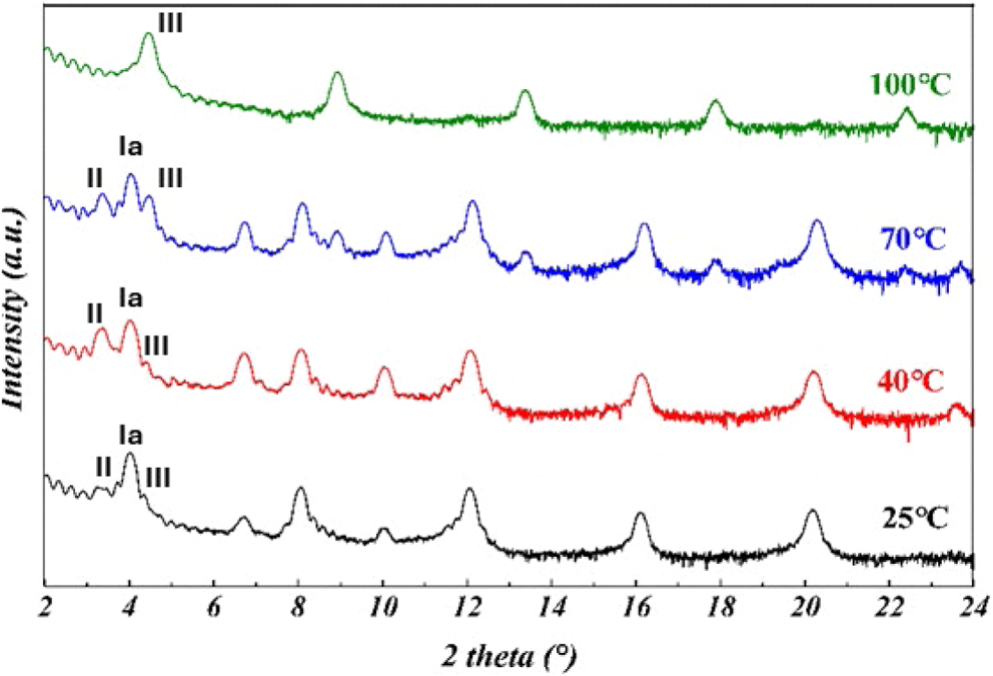
XRD patterns of ditbuc6-BTBT deposited at different substrate temperatures onto the Al_2_O_3_/TDPA substrate. The characteristic peak of each phase is indicated in the figure.

## Conclusions

In conclusion, we present a thorough study of the novel semiconductor ditBuC6-BTBT, emphasizing the wide polymorph screening conducted in bulk and thin films, which led to the discovery of four polymorphs: Form I, Ia, II, and III. While we determined the crystal structures of Form Ia and Form II, obtaining these structures was not straightforward due to the inherent challenges associated with polymorphism, including the difficulty in growing high-quality crystals suitable for structural analysis. The presence of the bulky group at the end of the alkyl chain led to a tilted core as observed in the ditBu-BTBT^39^ in contrast to the alkyl chain substituted BTBT. Notably, the structures exhibit a *Z*′ > 1, an unusual characteristic for symmetric BTBT molecules. When conducting the polymorph screening of Form I, we discovered that the recrystallization of the starting powder yielded the thermodynamically stable Form II, along with a distinct polymorph named Form Ia. We however noticed that both the metastable forms, Forms I and Ia, became dramatically unstable over time and converted to stable Form II due to an unintentional seeding effect. Consequently, Forms Ia and I can be classified as “disappearing polymorphs”. Form III is enantiotropically related to Forms I, Ia, and II, and it is stable at temperatures exceeding 138 °C. To expand the polymorph screening in view of the fabrication of devices, we explored the deposition of thin films by spin-coating, drop-casting, and nonconventional techniques for directional crystallization using thermal gradient and solution shearing. Nevertheless, in all the experiments, we only observed the concomitant bulk phases, and no thin-film phase or nonequilibrium phase was observed. While bulk studies made it easier to isolate each polymorph, thin-film deposition invariably resulted in concomitant polymorphs. Polymorph coexistence posed a significant challenge, especially for device performance. Despite efforts to isolate individual polymorphs, thin-film deposition methods consistently resulted in concomitant bulk phases, preventing the formation of a distinct thin-film phase. This coexistence impacted the charge carrier injection efficiency and contributed to poor device performance.

From the crystal structures of Form Ia and Form II, transfer integrals revealed 1D charge transport in Form II and 2D transport in Form Ia within the (0 0 1) plane. Furthermore, we fabricated bottom gate/bottom contact and bottom gate/top contact devices by using solution shearing and evaporation methods. Different parameters like temperatures, shearing speed, and the blend of ditBuC6-BTBT with polystyrene were also varied to improve mobility. We were able to extract moderate mobility values (μ_lin_ = 0.05 ± 0.01 cm^2^ V^–1^ s^–1^ and μ_sat_ = 0.03 ± 0.01 cm^2^ V^–1^ s^–1^) from the OFETs fabricated by evaporation at 70̊C. However, the deep ionization potential values of both polymorphs (Form Ia and Form II) led to inefficient charge carrier injection and the inability to completely isolate polymorphs further hindered electrical performance.

While the molecular design strategy in this study was not optimized for high-performance semiconductor applications, it provides valuable insights into the behavior of bulky-substituted BTBT systems, particularly with respect to polymorphism and crystallization challenges. Future work could focus on optimizing molecular design by exploring alternative substituents or modifying the bulky side chains to enhance crystallinity and better control polymorphic transitions. A better understanding of the relationship among molecular structure, polymorphism, and charge transport properties will be essential for designing high-performance materials for organic electronics.

## Supplementary Material



## Notes

Tables related to the solubility and polymorph screening, thermal gradient conditions, and ionization potentials along with the details and figures related to the purity assessment (NMR, HRMS, HPLC), TGA, DSC, HSM, PXRD, Pawley refinement, VTXRD, directional crystallization conditions, POM images, and transfer characteristics of OFET (PDF)

## ††

Laboratoire de Chimie des Polymères, Faculté des Sciences, Université Libre de Bruxelles (ULB), Boulevard du Triomphe, 1050 Bruxelles, Belgium

## Abbreviations

2MX: 2-methoxyethanol (2MX)
2PR: 2-propanol
ABZ: benzyl alcohol
ANI: anisole
BGBC: bottom gate/bottom contact
BGTC: bottom gate/top contact devices
CHF: chloroform
DCM: dichloromethane
DEC: diethyl carbonate
DFT: density functional theory
ditBuC6-BTBT: 2,7-bis­(7,7-dimethyloctyl)­benzo­[*b*]­benzo­[4,5]­thieno­[2,3-*d*]­thiophene
DMA: *N*,*N*-dimethylacetamide
DMF: *N*,*N*-dimethylformamide
DMS: dimethyl sulfoxide
DMX: 1,2-dimethoxyethane
DSC: differential scanning calorimetry
ETH: ethanol
HOMO: highest occupied molecular orbital
HPLC: high-performance liquid chromatography
HRMS: high-resolution mass spectrometry
HSM: hot stage microscopy
HT: high temperature
IE: ionization energies
IPA: isopropyl acetate
IPE: isopropyl ether
LUMO: lowest unoccupied molecular orbital
MEK: methyl ethyl ketone
MPY: 1-methyl-2-pyrrolidone
MYTHEN: Microstrip sYstem for TimerEsolved experimeNts
NMR: nuclear magnetic resonance
OFETs: organic field-effect transistors
OSCs: organic semiconductors
PFBT: pentafluorobenzenethiol
POM: polarized optical microscope
PS: polystyrene
PSI: Paul Scherrer Institute
PXRD: powder X-ray diffraction
PXY: *p*-xylene
PYS: photoemission yield spectroscopy
RT: room temperature
SAM: self-assembled monolayer
SCXRD: single-crystal X-ray diffraction
TDPA: *n*-tetradecylphosphonic acid
TGA-EGA: thermogravimetric analysis-evolved gas analysis
THF: tetrahydrofuran
TOL: toluene
VTXRD: in situ variable-temperature X-ray diffraction
