# Supplementary material for: Polymorph Screening and Investigation of Charge Transport of ditBuC6-BTBT
Source: Cryst Growth Des. 2025 Jun 3;25(12):4214–29. doi: 10.1021/acs.cgd.5c00046 (PMC12186265; doi:10.1021/acs.cgd.5c00046)
Supplement: Supplementary file 1 [file cg5c00046_si_001.pdf]

Supporting Information

Polymorph Screening and Investigation of Charge Transport of ditBuC6-BTBT

Priya Pandey[a,b,c]†, Federico Modesti[d], Nemo McIntosh[e], Christian Ruzié[c] Nicholas Turetta[f], Lamiaa Fijahi[g], Massimiliano Remigio[c], Guillaume Schweicher[c], Yves Henri Geerts[c,h,i], Marta Mas-Torrent[g], Peter Erk[d,j], Jérôme Cornil[e], Paolo Samorì[f], Enrico Modena[a]*, Lucia Maini[b]*

a PolyCrystalLine SPA, Via Della Cooperazione, 29 40059 Medicina, Bologna, Italy

b Dipartimento di Chimica "G. Ciamician", via Selmi 2 – Università di Bologna, I-40126, Bologna, Italy

c Laboratoire de Chimie des Polymères, Faculté des Sciences, Université Libre de Bruxelles (ULB), Boulevard du Triomphe, 1050 Bruxelles, Belgium

d BASF SE, Carl-Bosch-Straße 38, 67063 Ludwigshafen am Rhein, Germany

e Laboratory for Chemistry of Novel Materials, University of Mons, 7000 Mons, Belgium

f University of Strasbourg, CNRS, ISIS UMR 7006, 8 Alleé Gaspard Monge, Strasbourg, F-67000 France

g Institut de Ciència de Materials de Barcelona (ICMAB-CSIC), Campus de la UAB, 08193, Bellaterra, Spain

h International Solvay Institutes, Université Libre de Bruxelles (ULB), CP 231 Boulevard du Triomphe, 1050, Bruxelles, Belgium

i WEL Research Institute, avenue Pasteur, 6, 1300 Wavre, Belgium

j rConTec GmbH, Roter-Turm-Weg 3, 67157 Wachenheim an der Weinstraße, Germany

Table S 1 Solubility test results using 21 different solvents for 5 mg of ditBuC6-BTBT starting material powder. "X": suspension, "YES": clear solution, "-": not performed, and SS*: sparingly soluble.

	**Temperature**	**RT (20-25°C)**					**50°C**	**75°C**
	**Solvent**	**50μL**	**100μL**	**250μL**	**500μL**	**1mL**	**1mL**	**1mL**
	**Volume added to 5 mg**	**50μL**	**100μL**	**250μL**	**500μL**	**1mL**	**1mL**	**1mL**
	**Concentration (mg mL^{-1})**	**100**	**50**	**20**	**10**	**5**	**5**	**5**
2MX	2-Methoxyethanol	X	X	X	X	X	X	X
2PR	2-Propanol	X	X	X	X	X	X	Yes
ABZ	Benzyl Alcohol	X	X	X	X	X	X	Yes
ACN	Acetonitrile	X	X	X	X	X	X	X
ANI	Anisole	X	X	Yes	-	-	-	-
CHF	Chloroform	Yes	-	-	-	-	-	-
CLB	Chlorobenzene	Yes	-	-	-	-	-	-
DCM	Dichloromethane	X	X	X	X	X	Yes	-
DEC	Diethyl Carbonate	X	X	X	X	X	Yes	-
DMA	*N,N*-dimethylacetamide	X	X	X	X	X	Yes	-
DMF	*N,N*-dimethylformamide	X	X	X	X	X	X	X
DMF	Dimethyl Sulfoxide	X	X	X	Yes	-	-	-
ETH	Ethanol	X	X	X	X	X	X	SS*
H2O	Water	X	X	X	X	X	X	X
IPA	Isopropyl Acetate	X	X	X	X	Yes	-	-
IPE	Isopropyl Ether	X	X	X	X	Yes	-	-
MEK	Methyl ethyl Ketone	X	X	X	X	Yes	-	-
MPY	1-Methyl-2-Pyrrolidone	X	X	X	X	X	Yes	-
PXY	p-Xylene	X	Yes	-	-	-	-	-
THF	Tetrahydrofuran	Yes	-	-	-	-	-	-
TOL	Toluene	X	Yes	-	-	-	-	-

Table S 2 Polymorph screening experiments and the respective polymorphs obtained.

Experiment	Solvent/ Solvent mixture	Form I	Form II	Form I+Form II mixture
**Evaporation**	2-Propanol	-	-	Yes
	Benzyl Alcohol	-	-	Yes
	Anisole	Yes	-	-
	Chloroform	Yes	-	-
	Dichloromethane	Yes	-	-
	Diethyl Carbonate	-	Yes	-
	N,N-dimethylacetamide	-	-	Yes
	N,N-dimethylformamide	-	-	Yes
	1,2- Dimethoxyethane	-	Yes	-
	Isopropyl Acetate	-	-	Yes
	Isopropyl Ether	Yes	-	-
	Methyl ethyl Ketone	-	-	Yes
	1-Methyl-2-Pyrrolidone	-	-	Yes
	p-Xylene	Yes	-	-
	Tetrahydrofuran	Yes	-	-
	Toluene	Yes	-	-
	THF+Toluene (1:1)	-	-	Yes
**Anti-solvent addition (1:1)**	Acetonitrile: p-Xylene	Yes	-	-
	Chloroform: 2-Propanol	-	-	Yes
	Chloroform: Dimethyl Sulfoxide	Yes	-	-
	Tetrahydrofuran: Ethanol	Yes	-	-
	Toluene: Ethanol	-	-	Yes
**Slurry maturation**	2-Methoxyethanol			
	4-days	Yes	-	-
	7-days		-	-
	20-days		-	-
	Acetonitrile			
	4-days	-	-	Yes
	7-days	-	-	
	20-days	-	-	
	N,N-dimethylacetamide			
	4-days	-	-	Yes
	7-days	-	-	Yes
	20-days	-	Yes	-
	N,N-dimethylformamide			
	4-days	-	-	Yes
	7-days	-	-	Yes
	20-days	-	Yes	-
	Dimethyl Sulfoxide			
	4-days	Yes	-	-
	7-days		-	-
	20-days		-	-
	Ethanol			
	4-days	-	-	Yes
	7-days	-	-	Yes
	20-days	-	Yes	-
	Water			
	4-days	Yes	-	-
	7-days		-	-
	20-days		-	-
**Slurry maturation (Solvent mixtures)**	2-Methoxyethanol + *N,N*-dimethylformamide			
	4-days	Yes	-	-
	7-days		-	-
	20-days		-	-

	2-Methoxyethanol + 2-Propanol<br>4-days<br>7-days<br>20-days	-<br>-<br>-	Yes	-<br>-<br>-
	2-Methoxyethanol + 1,2-Dimethoxyethane (50°C)<br>4-days<br>7-days<br>20-days	-<br>-<br>-	Yes	-<br>-<br>-
	Acetonitrile + p-Xylene<br>4-days<br>7-days<br>20-days	-<br>-<br>-	Yes	-<br>-<br>-
	Toluene + Ethanol<br>4-days<br>7-days<br>20-days	-<br>-<br>-	Yes	-<br>-<br>-
**Crystal16: temperature gradient crystallization**	*N,N*-dimethylacetamide	Yes	-	-
	N,N-dimethylformamide	Yes	-	-
	Ethanol	-	Yes	-
**Mechanochemistry**	Dry grinding	Yes	-	-

Table S 3 Summary of the different gradient conditions applied in the present study.

**Entry**	**T_h (˚C)**	**T_c (˚C)**	**Pulling velocity *V* (μm s^{-1})**	**Gradient magnitude *G* (˚C mm^{-1})**	**Cooling rate (˚C s^{-1})**
**TG$_{111}$**	170	70	25	50	1.25
**TG$_{112}$**	170	70	50	50	2.5
**TG$_{121}$**	170	80	25	45	1.125
**TG$_{122}$**	170	80	50	45	2.25
**TG$_{131}$**	170	90	25	40	1
**TG$_{132}$**	170	90	50	40	2
**TG$_{141}$**	170	100	5	35	0.175
**TG$_{142}$**	170	100	10	35	0.35
**TG$_{143}$**	170	100	25	35	0.875
**TG$_{144}$**	170	100	50	35	1.75
**TG$_{151}$**	170	120	25	25	0.625
**TG$_{152}$**	170	120	50	25	1.25
**TG$_{161}$**	170	140	5	15	0.075
**TG$_{162}$**	170	140	10	15	0.15
**TG$_{163}$**	170	140	20	15	0.3
**TG$_{164}$**	170	140	25	15	0.375
**TG$_{165}$**	170	140	50	15	0.75
**TG$_{211}$**	180	140	5	20	0.1
**TG$_{212}$**	180	140	10	20	0.2
**TG$_{213}$**	180	140	25	20	0.5
**TG$_{214}$**	180	140	50	20	1

Table S 4 Ionization energy (IE) values of ditBuC6-BTBT as determined by PYS from powder (IEp) and single-crystalline (IESC) samples. SC samples lay on the substrate with the short axis perpendicular to the substrate plane.

Compound	IE_p (eV)	IE_{SC} (eV)
ditBuC6-BTBT (I)	5.78 ± 0.03	5.81 ± 0.05
ditBuC6-BTBT (II)	5.57 ± 0.03	5.51 ± 0.05
C7-BTBT-C7	5.28 ± 0.02	-
diC7tBu-BTBT	5.59 ± 0.04	-

Experimental section-

Synthesis.
7,7-dimethyloctanoic acid (**1**) and benzo[b]benzo[4,5]thieno[2,3-d]thiophene (**3**) are known from the literature.[1,2] (Scheme S1)

Synthesis of neo-decanoic acid chloride

To a solution of neo-decanoic acid (14.59 g, 84.6 mmol) in dry CH_2Cl_2 (150 mL) was added dropwise in about 30 min oxalyl chloride (10.17 mL, 118 mmol) under argon atmosphere. The reaction mixture was stirred further for 2h, and then evaporated to dryness. Neo-decanoic acid chloride was directly used in the next reaction.

Synthesis of 1,1'-(benzo[b]benzo[4,5]thieno[2,3-d]thiophene-2,7-diyl)bis(7,7-dimethyloctan-1-one), ditBuC$_5$CO-BTBT
To a solution of [1]benzothieno[3,2-*b*][1]thiophene BTBT (4.07 g, 16.93 mmol) in dry CH_2Cl_2 (400 mL) was added $AlCl_3$ (11.29 g, 84.67 mmol) in one portion, at –20 °C and under an argon atmosphere. The reaction mixture was stirred for 15 min and then was cooled down to –78 °C. neo-decanoic acid chloride was then added dropwise in about 1 h. The reaction mixture was stirred at –78 °C for 2h and allowed to warm to room temperature. After 2 days at room temperature, the reaction was quenched by the addition of ice/water (100 mL). Volatiles were removed under reduced pressure. The formed precipitate was isolated by filtration, washed with water (3x50 mL) and methanol (3x50 mL) and finally dried to afford a yellow powder. Further recrystallization in toluene afforded a yellow crystalline powder (3.84 g, 41 %). ^{1}H NMR (400 MHz, $CDCl_3$, 25°C) δ = 8.56 (dd, ^4J(H,H) = 1.5 Hz and ^5J(H,H) = 0.6 Hz , 2 H, CH), 8.07 (dd, ^3J(H,H) = 8.4 Hz and ^4J(H,H) = 1.5 Hz, 2 H, CH), 7.96 (dd, ^3J(H,H) = 8.4 Hz and ^5J(H,H) = 0,6 Hz, 2 H, CH), 3.10-3.04 (m, 4H, CH_2), 1.85-1.75 (m, 4H, CH_2), 1.42-1.27 (m, 8 H, CH_2), 1.20-1.16 (m, 4 H, CH_2), 0.88 (s, 18H, CH_3); ^{13}C NMR (101 MHz, $CDCl_3$, 25°C) δ = 199.5 (C), 142.9 (C), 136.3 (C), 135.9 (C), 134.4 (C), 125.0 (CH), 124.7 (CH), 121.7 (CH), 44.2 (CH_2), 39.0 (CH_2), 30.4 (C)., 30.4 (CH_2), 29.5 (CH_3)., 24.6 (CH_2), 24.5 (CH_2); HRMS (Maldi-TOF): *m/z* calc for $C_{34}H_{44}O_2S_2$: 548.2783 ; found 548.2783.

Synthesis of 2,7-bis(7,7-dimethyloctyl)benzo[b]benzo[4,5]thieno[2,3-d]thiophene, ditBuC$_6$-BTBT:

A mixture of *ditBuC$_5$CO-BTBT* (2.74 g, 5.0 mmol), potassium hydroxide (1.40 g, 25.0 mmol) and hydrazine monohydrate (6.1 ml, 125.0 mmol) in diethylene glycol (300 ml) was heated to 120 °C for 1 h and to 240 °C overnight. After cooling to room temperature, the formed precipitate was filtered off and washed with methanol (2 × 15 ml). Column chromatography on silica gel (hexane). and crystallization from hexane afforded 1.77 g (68%) of ditBuC$_6$-BTBT. m.p.: 145 °C; ^{1}H NMR (400 MHz, CD_2Cl_2, 25°C) δ = 7.77 (d, ^1J(H,H) = 8.1 Hz, 2 H, CH), 7.74 (d, ^2J(H,H) = 1.0 Hz, 2 H, CH), 7.30 (dd, ^1J(H,H) = 8.1 Hz and ^2J(H,H) = 1.6 Hz, 2 H, CH), 2.77 (t, ^3J(H,H) = 8.0 Hz, 4H, CH_2), 1.73-1.66 (m, 4H, CH_2), 1.45-1.12 (m, 16H, CH_2), 0.87 (s, 18H, CH_3); ^{13}C NMR (101 MHz, $CDCl_3$, 25°C) δ = 142.40 (C), 140.43 (C), 132.52 (C), 131.13 (C), 125.99 (CH), 123.36 (CH), 120.99 (CH), 44.26 (CH_2), 36.09 (CH_2),

[1] Copper-Catalyzed Cross-Coupling of Functionalized Alkyl Halides and Tosylates with Secondary and Tertiary Alkyl Grignard Reagents,
Peng Ren, Lucas-Alexandre Stern, and Xile Hu, *Angew. Chem. Int. Ed.* **2012**, *51*, 9110-9113
[2] One-step synthesis of [1]benzothieno[3,2-*b*][1]benzothiophene from *o*-chlorobenzaldehyde, M. Saito, I. Osaka, E. Miyazaki, K. Takimiya, H. Kuwabara, and M. Ikeda, *Tetrahedron Lett.* **2011**, *52*, 285-288.

31.76 (CH_2), 30.55 (CH_2), 30.15 (C), 29.99 (CH_2), 29.40 (CH2), 29.18 (CH_3), 24.53 (CH_2); HRMS (Maldi-TOF): *m/z* calc for $C_{34}H_{48}S_2$: 520.3197 ; found : 520.3201.

; HRMS ( ): *m/z* calcd for $C_{34}H_{48}S_2$: ; found.

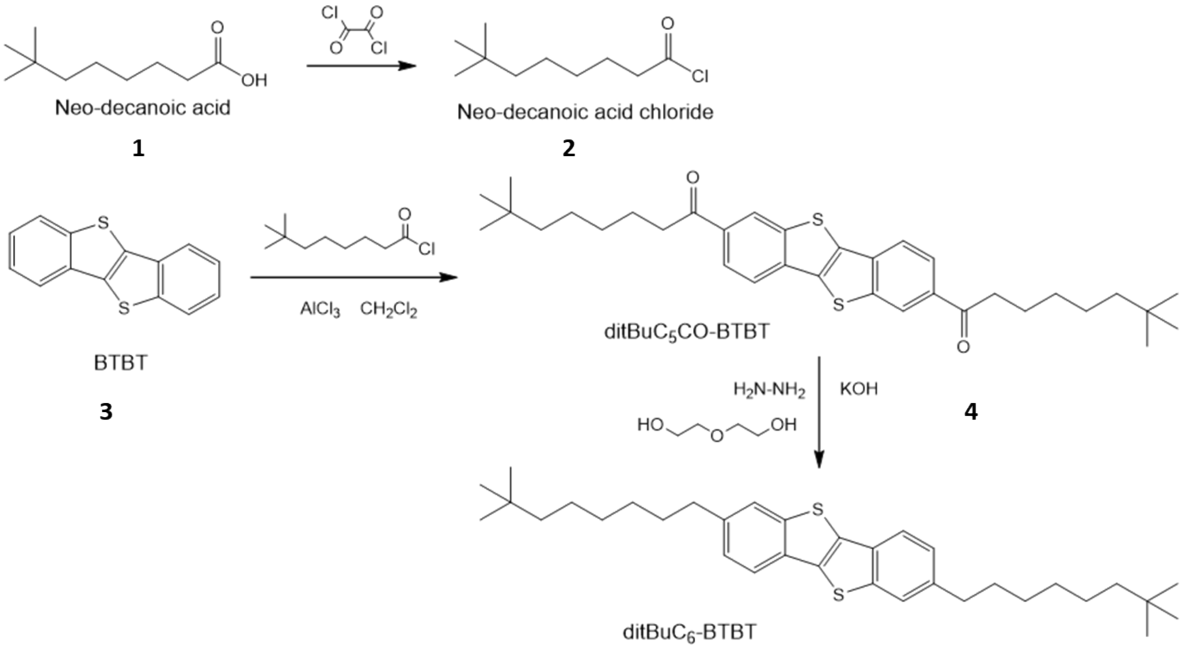

Scheme S 1 Synthesis of ditBuC6-BTBT

Nuclear magnetic resonance (NMR).

The ^{1}H NMR and 13C-NMR spectra were recorded at 298 K on a Jeol 400 MHz (J400) Royal probe spectrometer. Chemical shifts are reported in parts per million (ppm) with TMS as the internal standard. The concentration of the solution in $CDCl_3$ is 9.8 mg mL^{-1}, and the number of scans was 32.

Mass spectrometry.

Mass spectrometry analyses were conducted at the Organic Synthesis & Mass Spectrometry Laboratory, University of Mons, under the supervision of Prof. Pascal Gerbaux using MALDI-MS on a Q-TOF Premier mass spectrometer (Waters, Manchester, UK)

High-performance liquid chromatography (HPLC).

An Agilent HP 1100 system equipped with an HPLC column (Normal Phase, chiral, covalent bunded (Pirkle Covalent (R, R) Whelko-01 10/100 FEC 25cm x 4.6 mm)) and a UV detector were used to assess the purity of the analysed materials. Approximately 1 mg of the sample was dissolved in 1 mL of a mobile phase consisting of Hex-IPA 2:8 isocratic condition (both HPLC grade). The column temperature was set to 25˚C, and the chromatograms were analysed at 254 nm (see Figure S9).

Sublimation.

Severn Thermal oven equipped with TF50/7.5/3Z/F furnace (control unit: CU3Z330425660) was used to perform sublimation at 140˚C under 2.5 x 10^{-7} atm pressure.

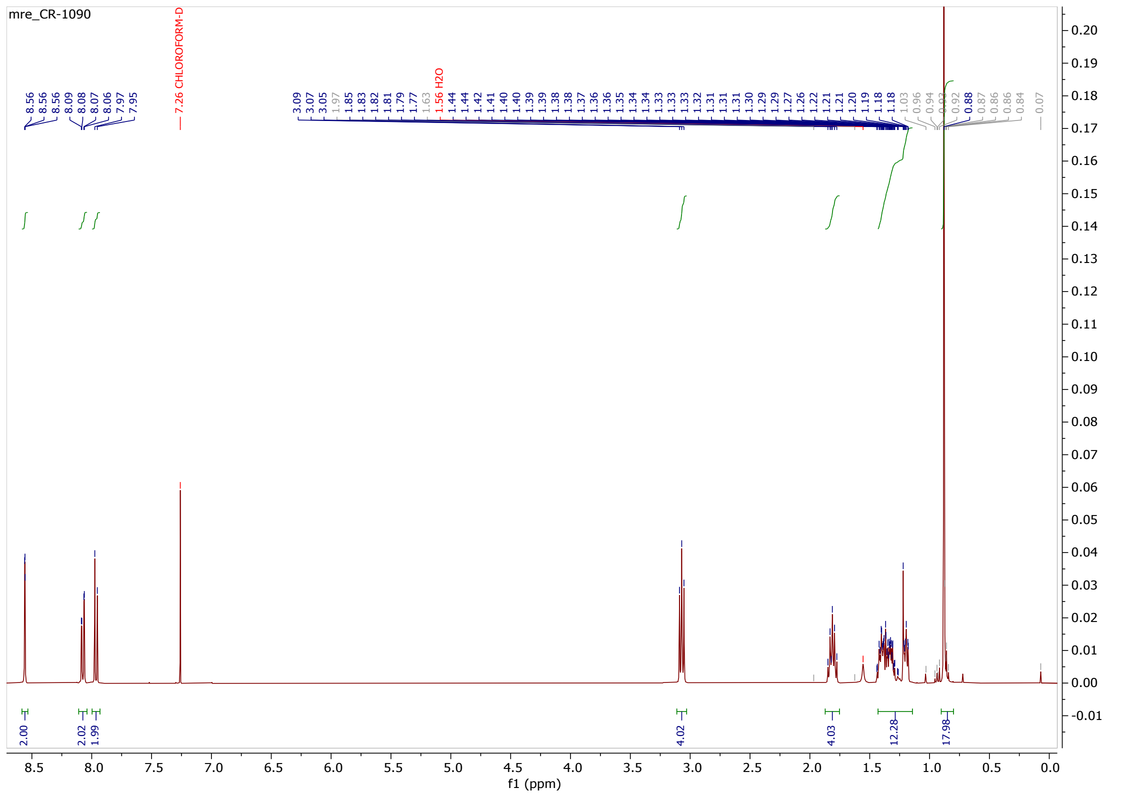

Figure S 1 ^{1}H NMR of ditBuC5CO-BTBT **(4)**

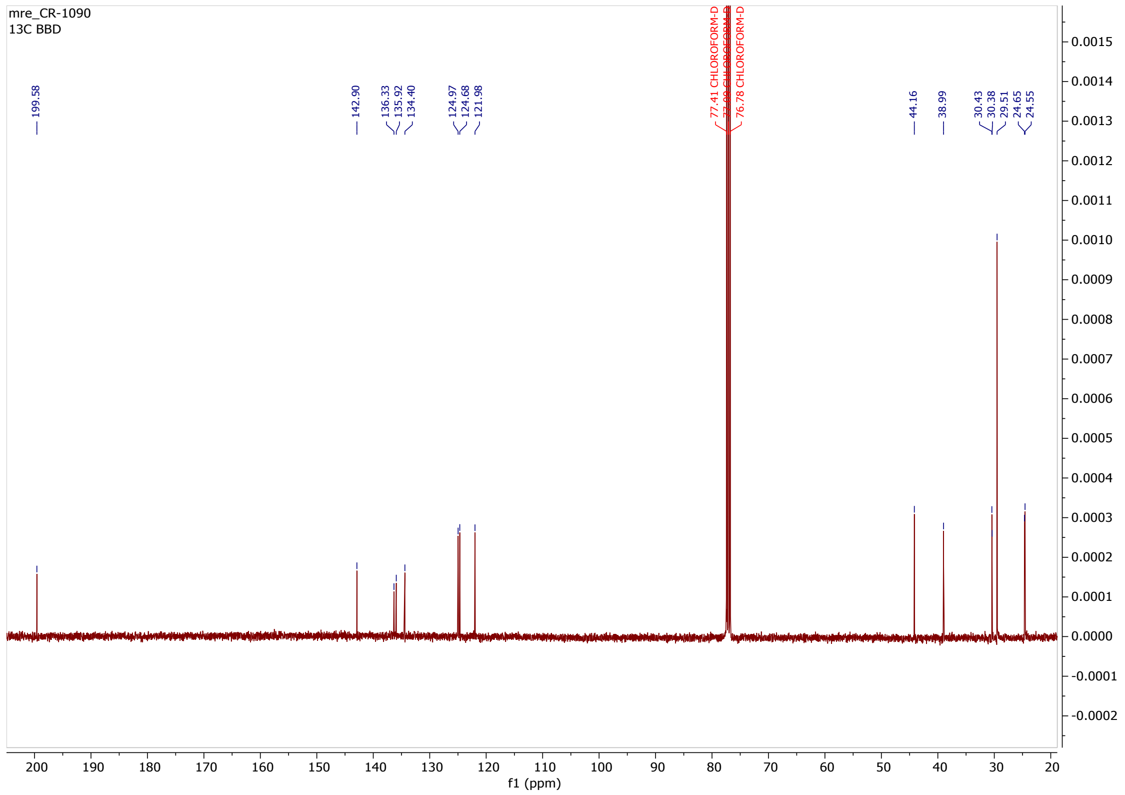

Figure S 2 13C- NMR of ditBuC5CO-BTBT **(4)**

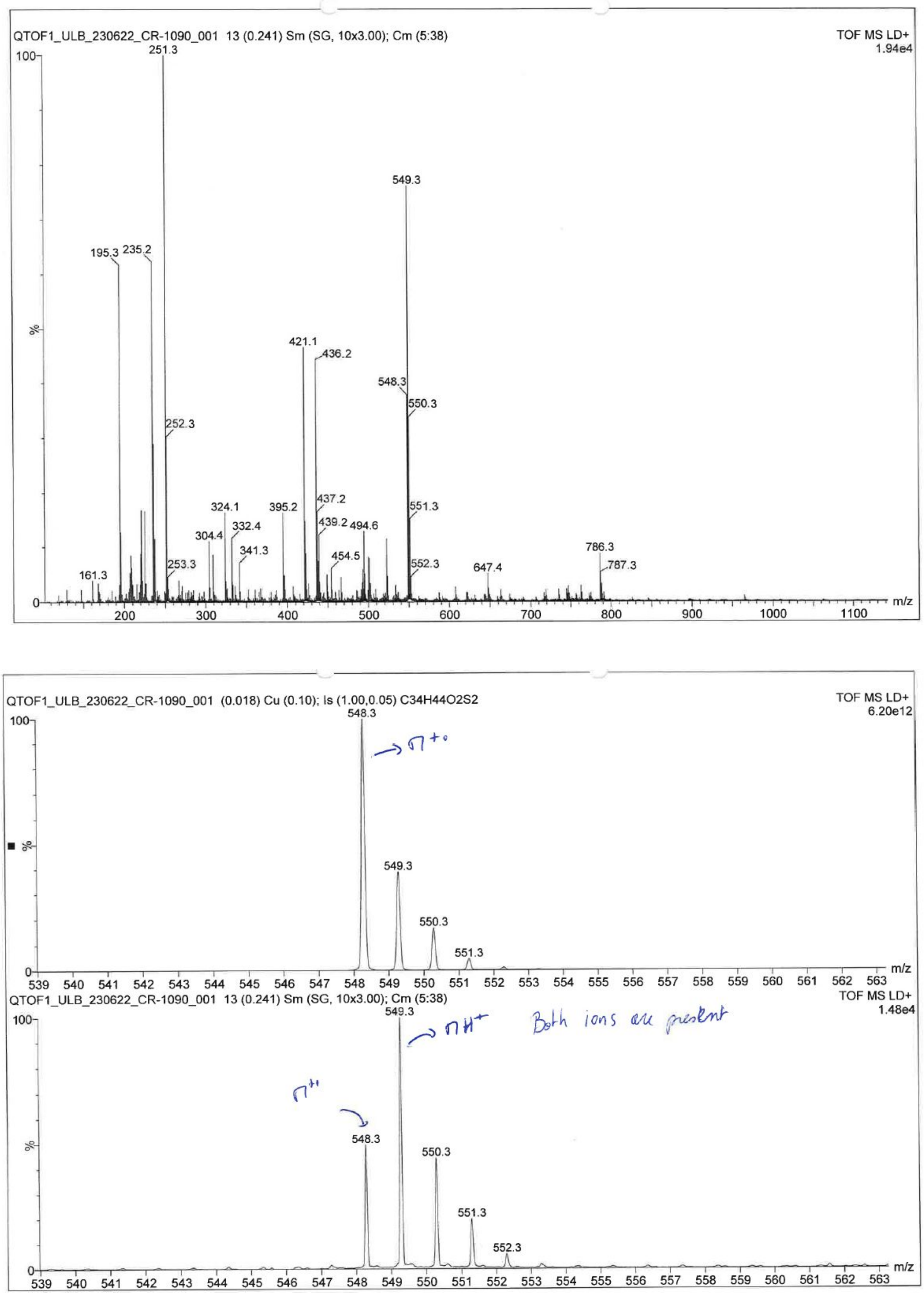

Figure S 3 High-resolution mass spectrometry (HRMS) spectra of ditBuC5CO-BTBT **(4)**

Elemental Composition Report | Page 1

Single Mass Analysis
Tolerance = 600.0 PPM / DBE: min = -1.5, max = 100.0
Element prediction: Off
Number of isotope peaks used for i-FIT = 3

Monoisotopic Mass, Odd and Even Electron Ions
6 formula(e) evaluated with 6 results within limits (all results (up to 1000) for each mass)
Elements Used:
C: 0-100 H: 0-200 O: 2-2 S: 2-2
QTOF1_ULB_230622_CR-1090_001 13 (0.241) AM (Med,3, Ar,9317.0,569.31,0.70); Sm (SG, 10x3.00); Cm ((9:15+49:70))
TOF MS LD+

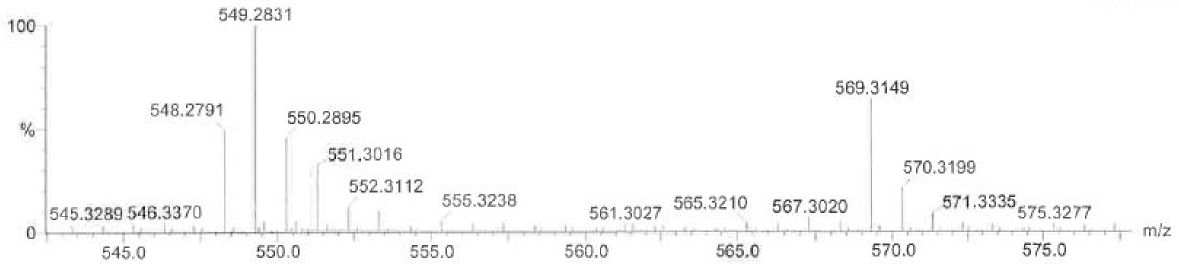

Mass	Calc. Mass	mDa	PPM	DBE	i-FIT	i-FIT (Norm)	Formula
Minimum:				-1.5			
Maximum:		5.0	600.0	100.0			
548.2791	548.2783	0.8	1.5	13.0	181.9	0.6	C34 H44 O2 S2
	548.3722	-93.1	-169.8	6.0	182.1	0.8	C33 H56 O2 S2
	548.1844	94.7	172.7	20.0	197.7	16.4	C35 H32 O2 S2
	548.4661	-187.0	-341.1	-1.0	200.2	18.9	C32 H68 O2 S2
	548.0905	188.6	344.0	27.0	217.4	36.1	C36 H20 O2 S2
	547.9966	282.5	515.2	34.0	216.4	35.1	C37 H8 O2 S2

Figure S 4 Elemental composition report of ditBuC5CO-BTBT **(4)**

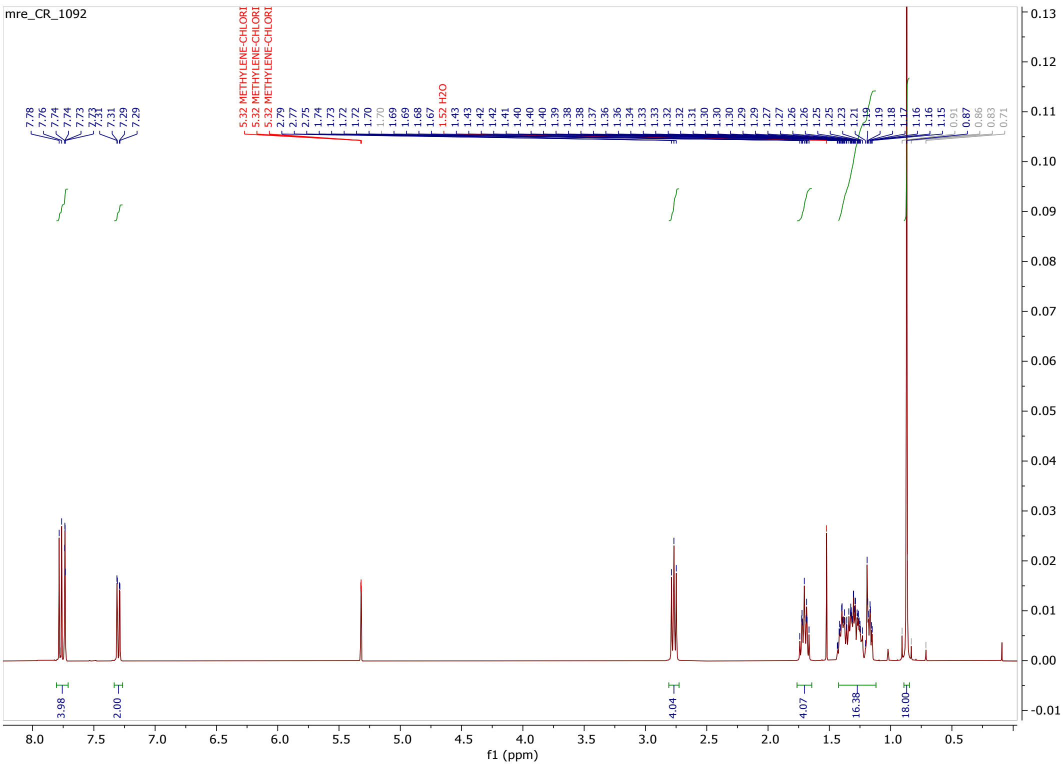

Figure S 5 ^{1}H NMR of ditBuC6-BTBT **(5)**

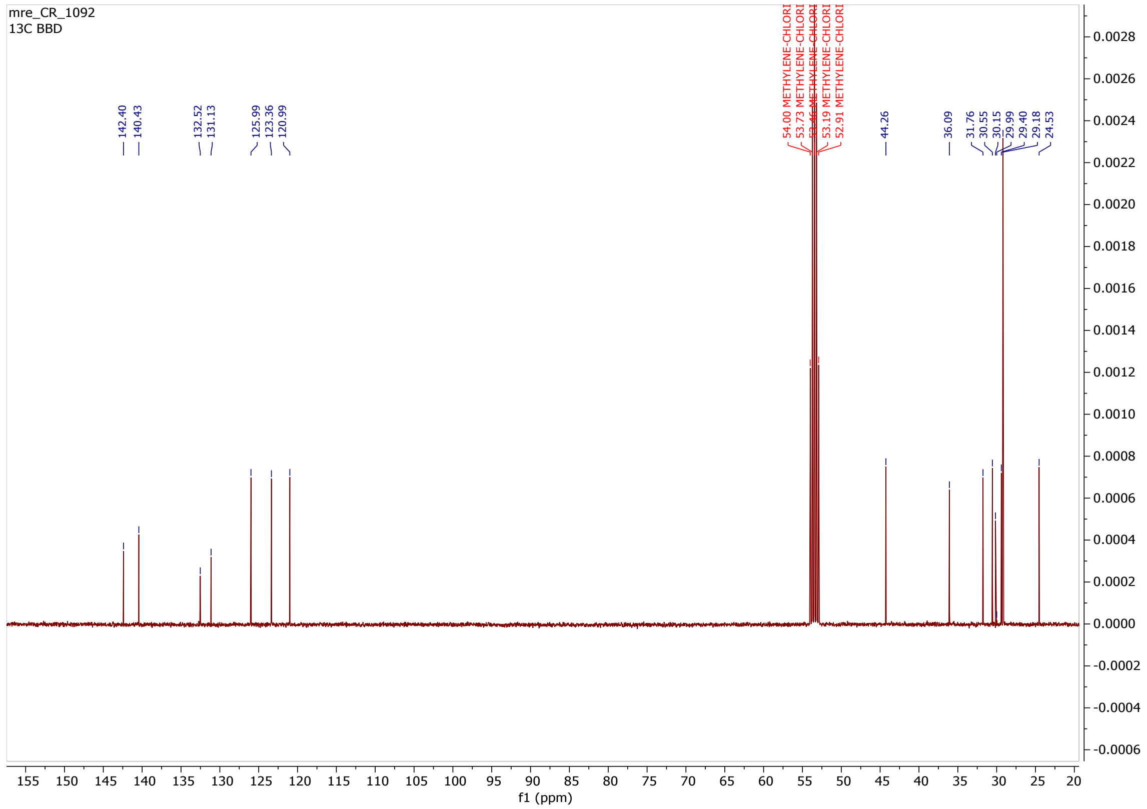

Figure S 6 13C- NMR of ditBuC6-BTBT **(5)**

Purity assessment of ditBuC6-BTBT.

The purity of ditBuC6-BTBT was assessed by ^{1}H NMR AND 13C-NMR. The sample was observed to be pure and no extra peaks were observed besides the expected water and $CDCl_3$ peaks (Figure S5). Therefore, the possibility of major contamination by another organic compound was ruled out. Further purity analysis with HPLC was performed to evaluate if any impurities that are lower than 5-6 % in weight are present. HPLC results showed two peaks with retention times 8.54 ± 0.01 min (area: 0.3 ± 0.1%) and 10.16 ± 0.02 min (area 99.6 ± 0.3%) (Figure S9a). We attempted to purify the compound by TLC using multiple solvent systems; however, the low concentration of the impurity and the close retention time between the impurity and the target molecule made it impossible to separate them by TLC. Thus, we performed sublimation of the material to purify it further, and post-sublimation HPLC resulted in only one peak with a retention time of 10.67 ± 0.02 min (Figure S9b). To analyse the phase of the pure compound, PXRD was performed.

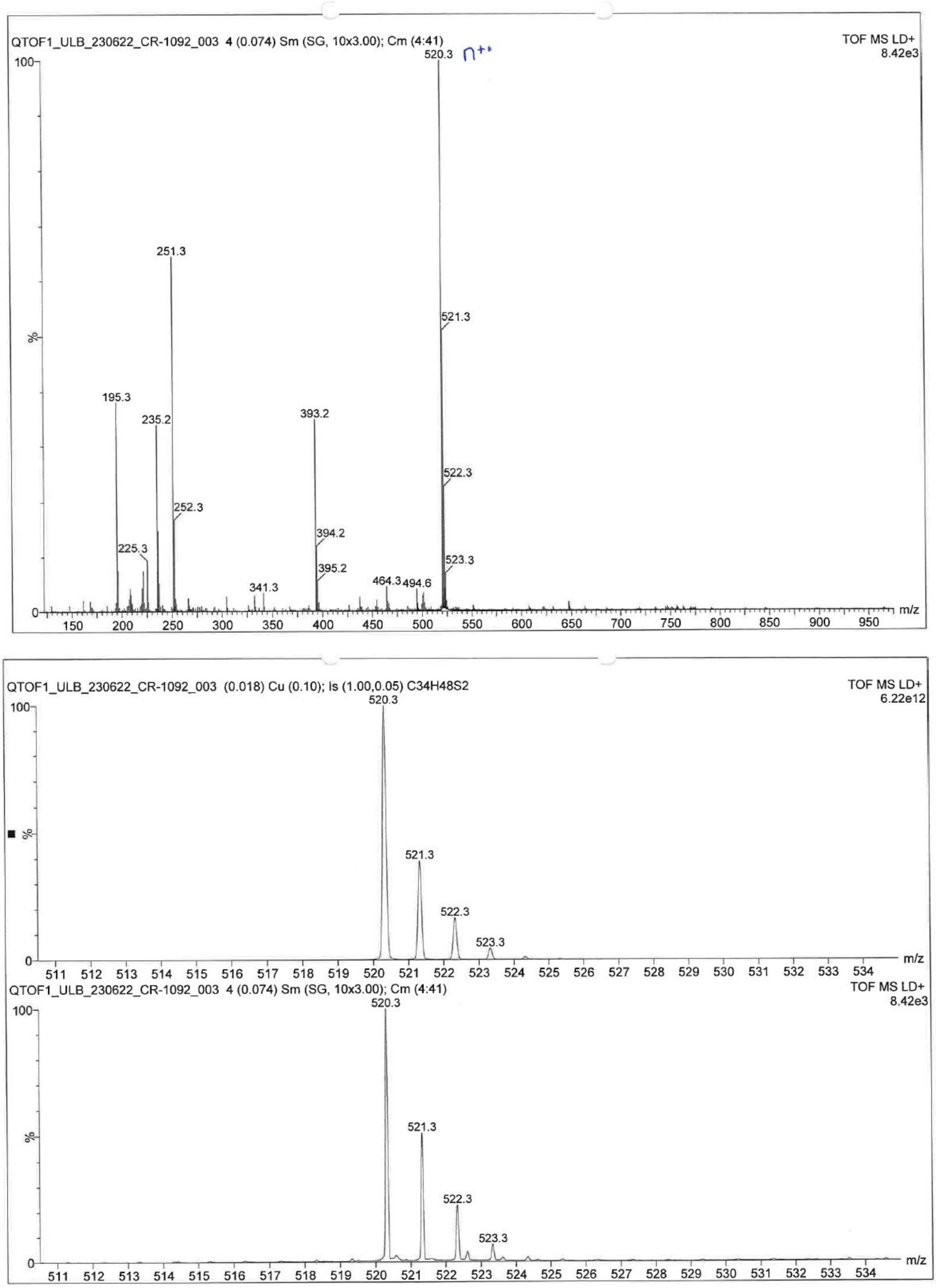

Figure S 7 HRMS spectra of ditBuC6-BTBT **(5)**

Elemental Composition Report Page 1

Single Mass Analysis
Tolerance = 600.0 PPM / DBE: min = -1.5, max = 100.0
Element prediction: Off
Number of isotope peaks used for i-FIT = 3

Monoisotopic Mass, Odd and Even Electron Ions
7 formula(e) evaluated with 5 results within limits (all results (up to 1000) for each mass)
Elements Used:
C: 0-100 H: 0-200 S: 2-2
QTOF1_ULB_230622_CR-1092_003 63 (1.167) AM (Med,3, Ar,9317.0,525.29,0.70); Sm (SG, 10x3.00); Sm (SG, 10x3.00); Cm (22:73)
TOF MS LD+

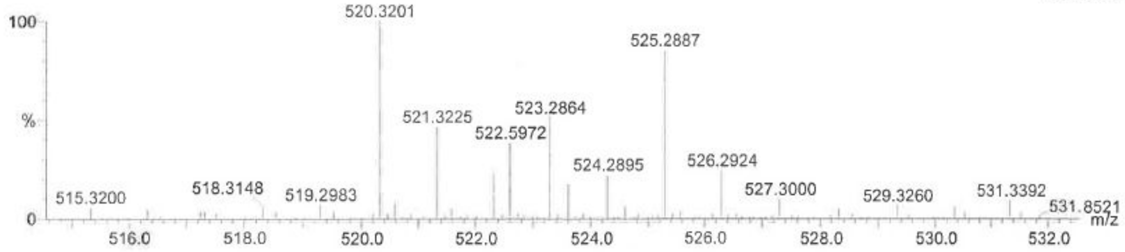

Minimum:				-1.5			
Maximum:		5.0	600.0	100.0			
Mass	Calc. Mass	mDa	PPM	DBE	i-FIT	i-FIT (Norm)	Formula
520.3201	520.3197	0.4	0.8	11.0	175.2	0.9	C34 H48 S2
	520.4136	-93.5	-179.7	4.0	175.1	0.9	C33 H60 S2
	520.2258	94.3	181.2	18.0	176.0	1.8	C35 H36 S2
	520.1319	188.2	361.7	25.0	221.8	47.5	C36 H24 S2
	520.0380	282.1	542.2	32.0	218.6	44.3	C37 H12 S2

Figure S 8 Elemental composition report of ditBuC6-BTBT **(5)**

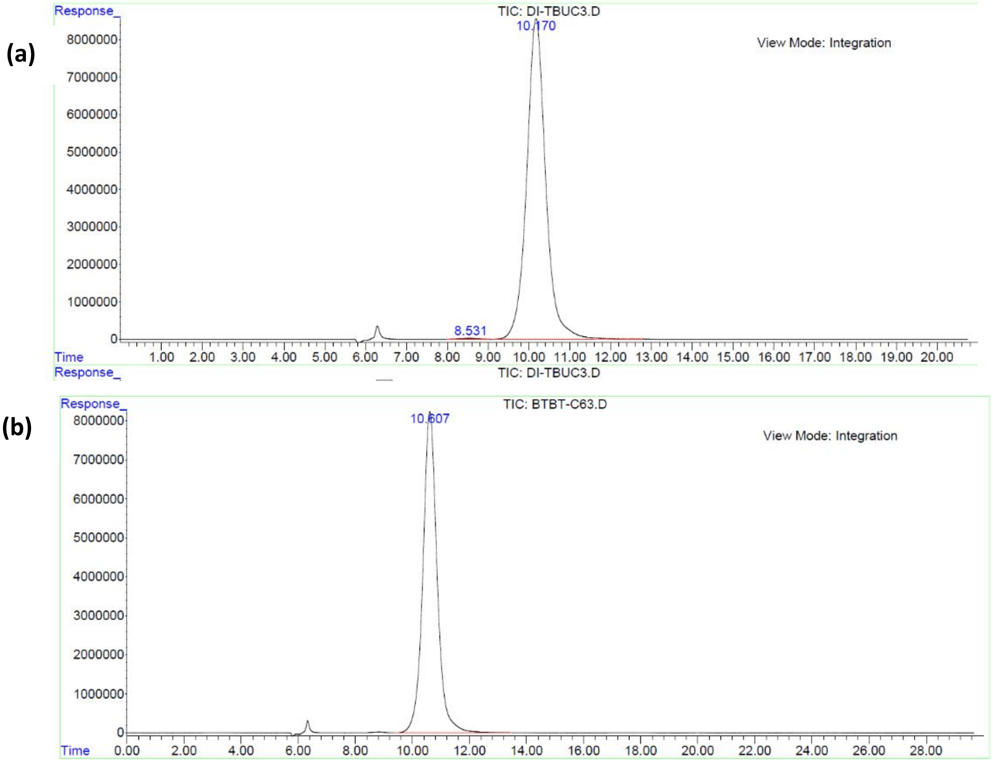

Figure S 9 High performance liquid chromatography (HPLC) spectra of ditBuC6-BTBT sample (a) before and (b) after sublimation.

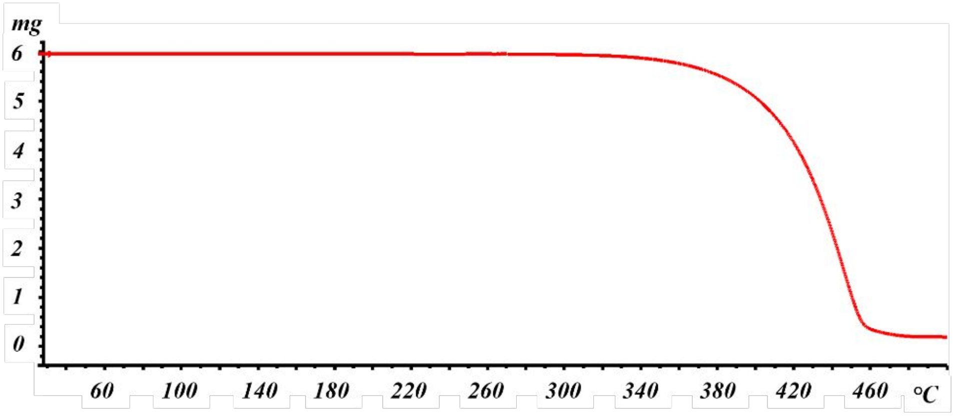

Figure S 10 Thermo Gravimetric analysis (TGA) of ditBuC6-BTBT at 10˚C min^{-1}.

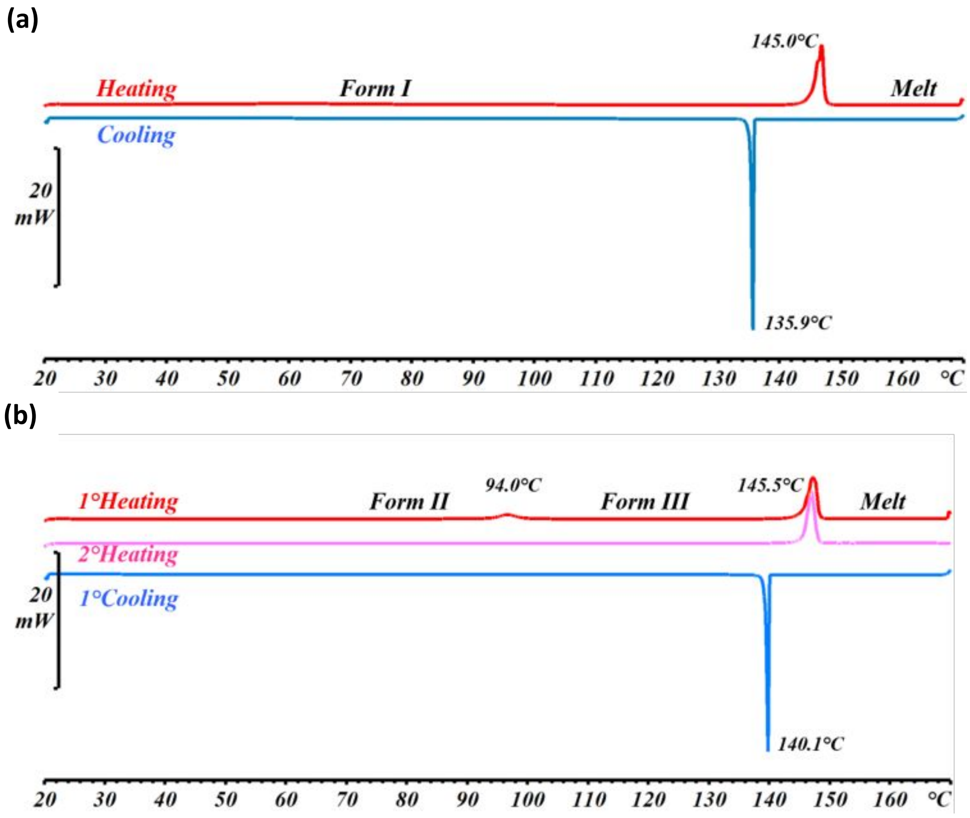

Figure S 11 Differential scanning calorimetry (DSC) of ditBuC6-BTBT of (a) Form I and (b) Form II, at 5˚C min^{-1}.

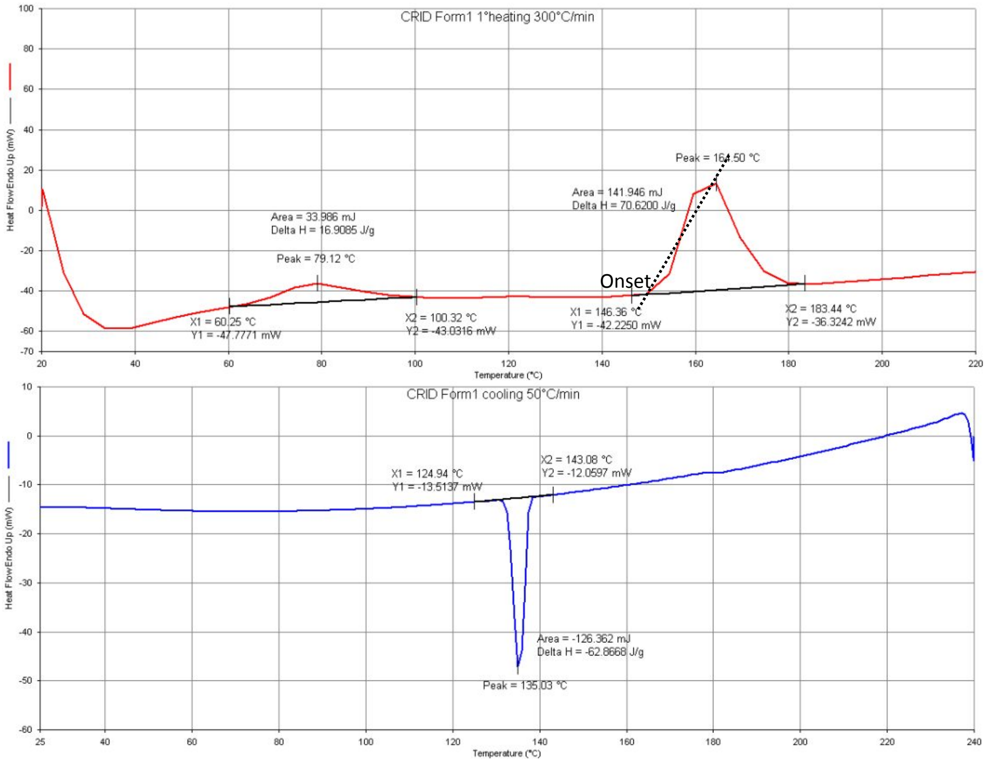

Figure S 12 DSC of Form I at 300˚C min^{-1}, showing the transition to Form III at T_{max}= 79˚C, the melting is observed with a T_{onset} ~ 150°C. The T_{max} is highly influenced by the rate of heating.

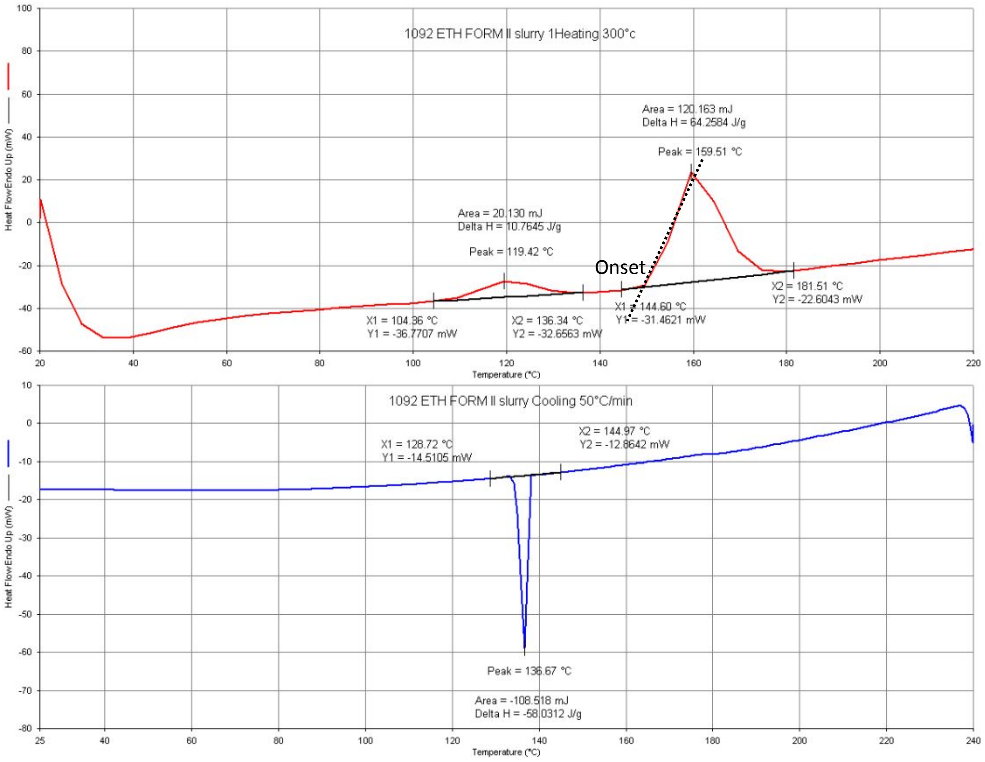

Figure S 13 DSC of Form II at 300˚C min^{-1}, showing the transition to Form III at T_{max}= 119˚C, the melting is observed with a T_{onset} ~ 150°C. The T_{max} is highly influenced by the rate of heating.

From all the three DSC figures S11, S12, and S13, it appears that the transition from Form I→Form III and Form II→Form III is irreversible. However, during the stability analysis we observed that Form III is only stable at high temperatures, as the slow conversion to Form I and then later to Form II was observed using XRD. The kinetics of transformation is very slow and thus undetected in DSC. Refer to Figure S28.

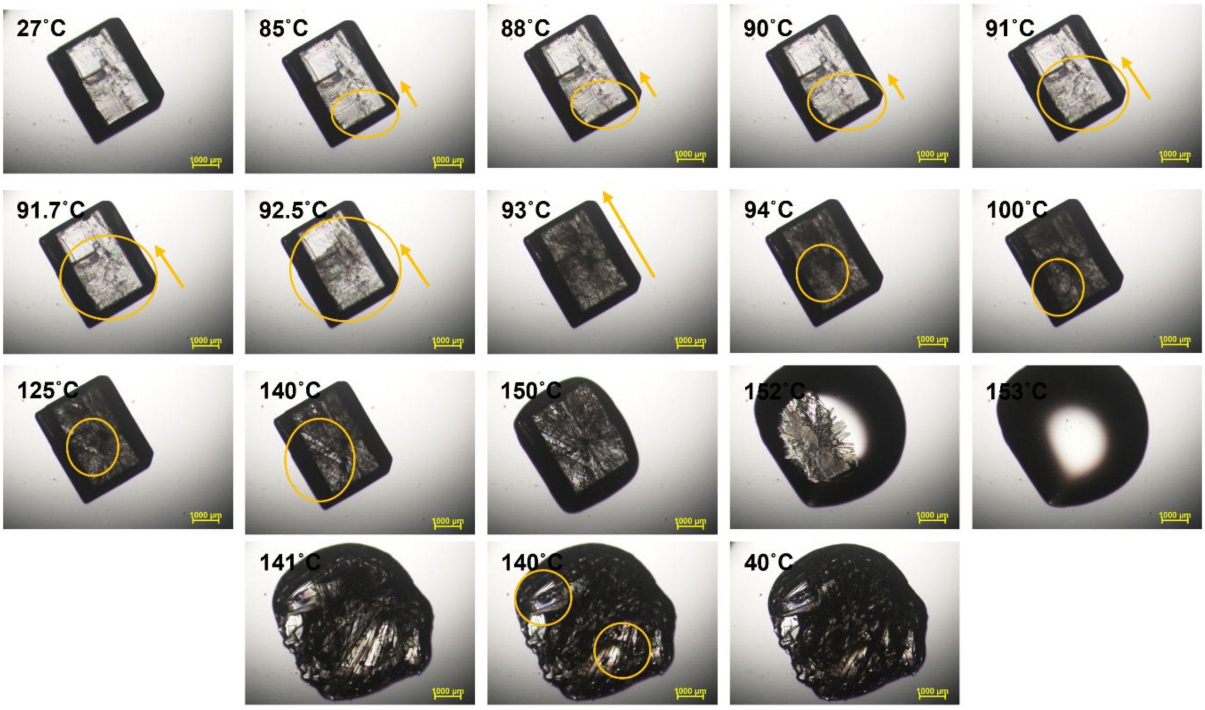

Figure S 14 Hot-stage microscope (HSM) images of crystal of Form Ia crystal, showing the transition starting from the bottom edge of the crystal at 85˚C and propagating to the top edge and complete transition to Form III at 140˚C, followed by melting at 153˚C. Recrystallization from melt occurred at 141˚C, further small changes were observed throughout cooling until 40˚C. Scale bar 1000 µm.

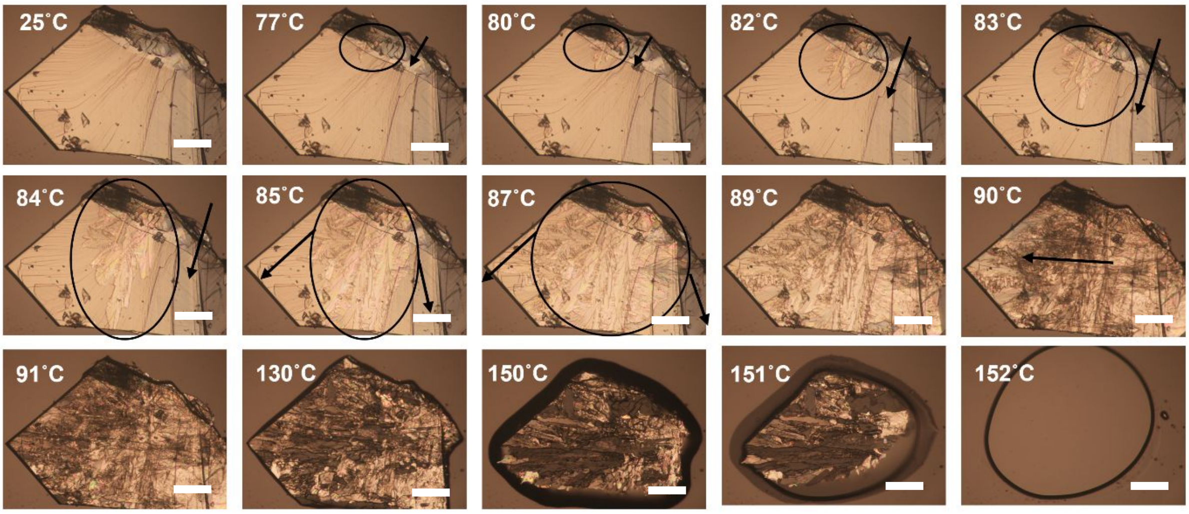

Figure S 15 HSM images of crystal of Form Ia crystal, showing the transition starting from the top edge of the crystal at 77˚C and propagating to the bottom edge and complete transition to Form III at 130˚C, followed by melting at 152˚C. Scale bar 500 µm.

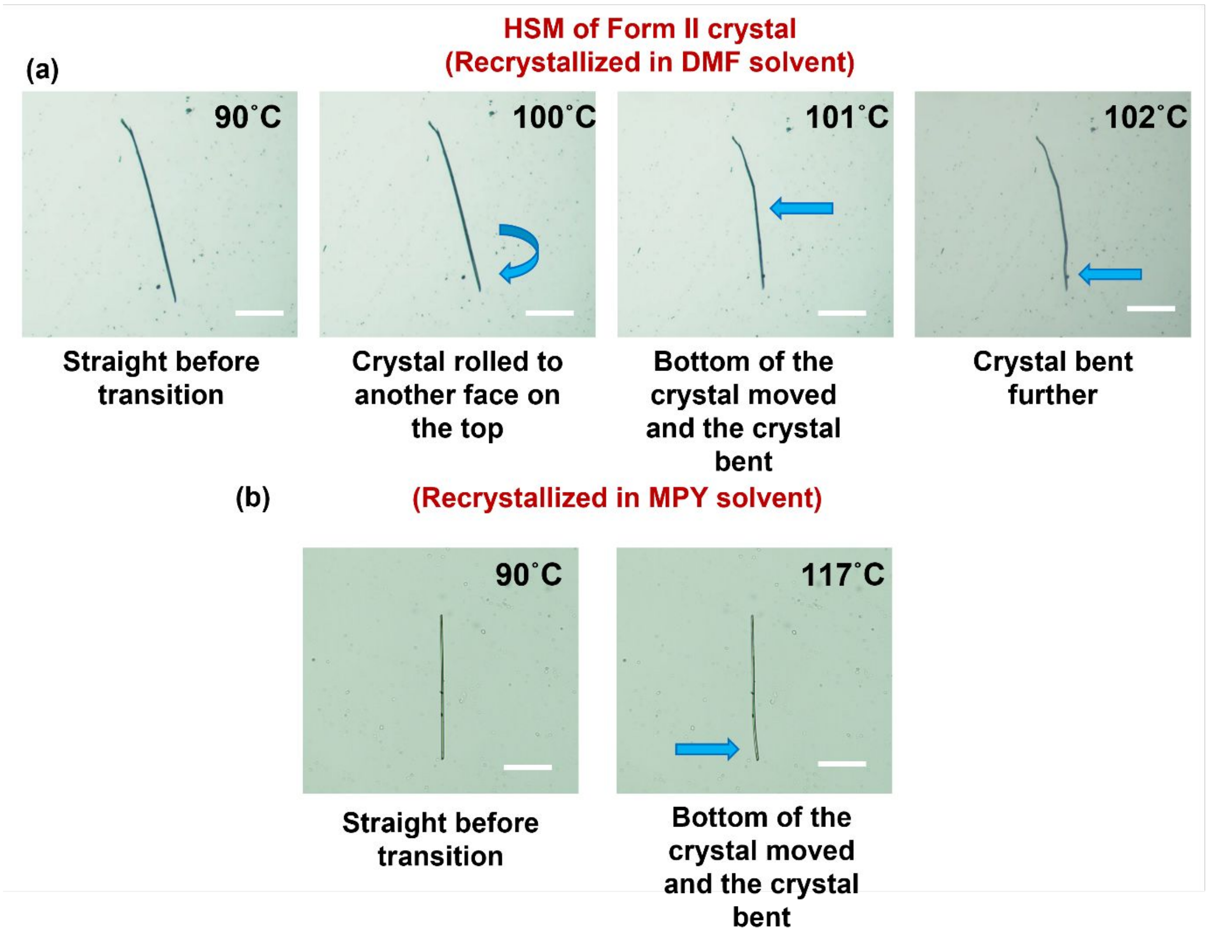

Figure S 16 HSM images of Form II crystals in (a) DMF solvent and (b) MPY solvent, showing the transition to Form III by slight movement at the bottom of the needle crystals. Scale bar 500 µm.

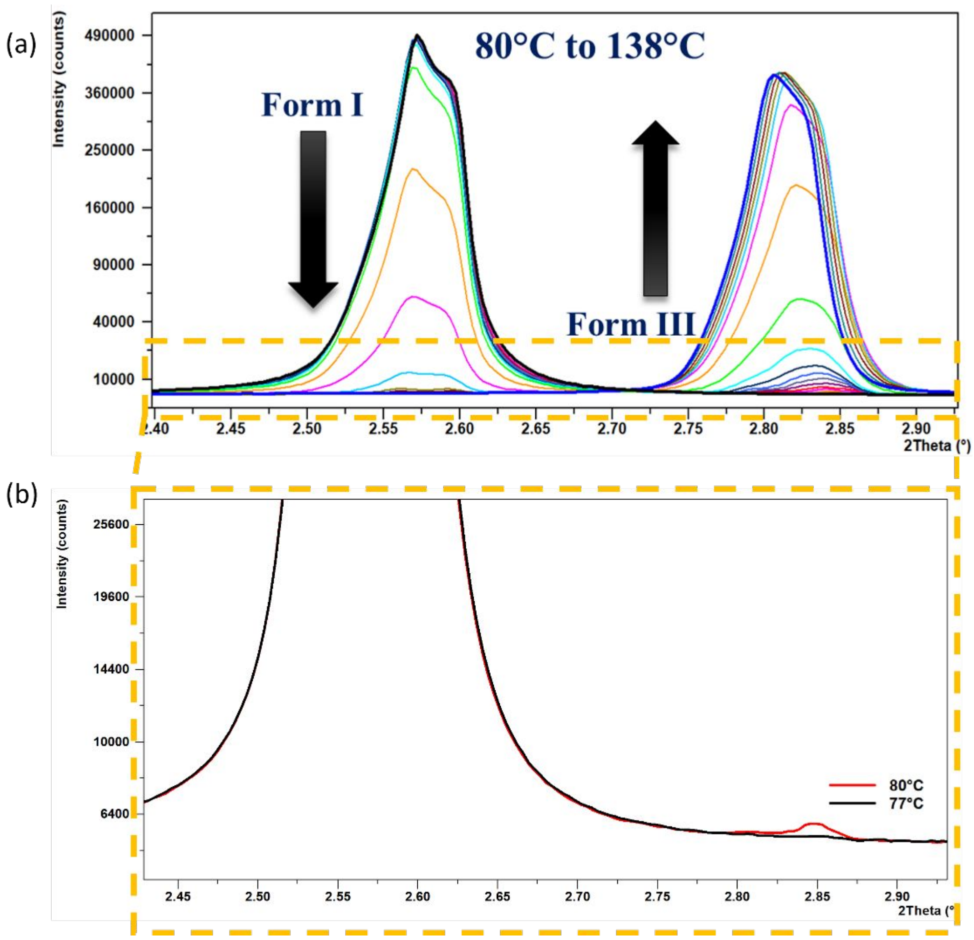

Figure S 17 (a) VTXRD of ditBuC6-BTBT from 77˚C (black line) to 138˚C (blue line), highlighting the gradual transition. A distinct peak corresponding to Form III begins to appear at 80 °C (λ = 0.9999613 Å). To emphasize the transformation, only the low-angle region of the diffractogram is shown. (b) Enlarged view of the relevant XRD region, illustrating the emergence of the Form III peak at 80 °C.

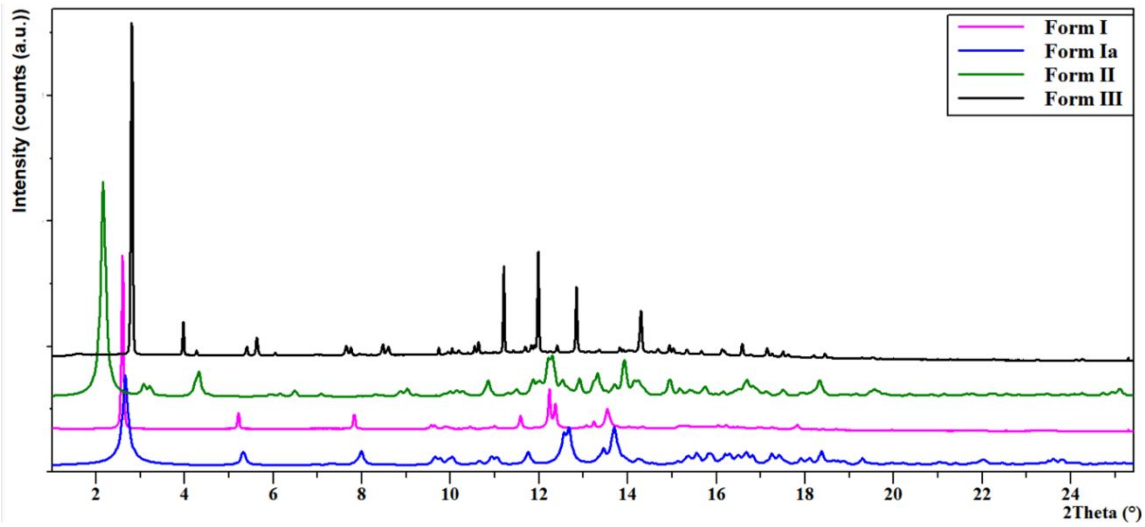

Figure S 18 Experimental powder X-ray diffraction (PXRD) patterns of all the polymorphs at λ= 0.9999613 Å. Form I, Ia, and II were collected at room temperature, and Form III at 138˚C.

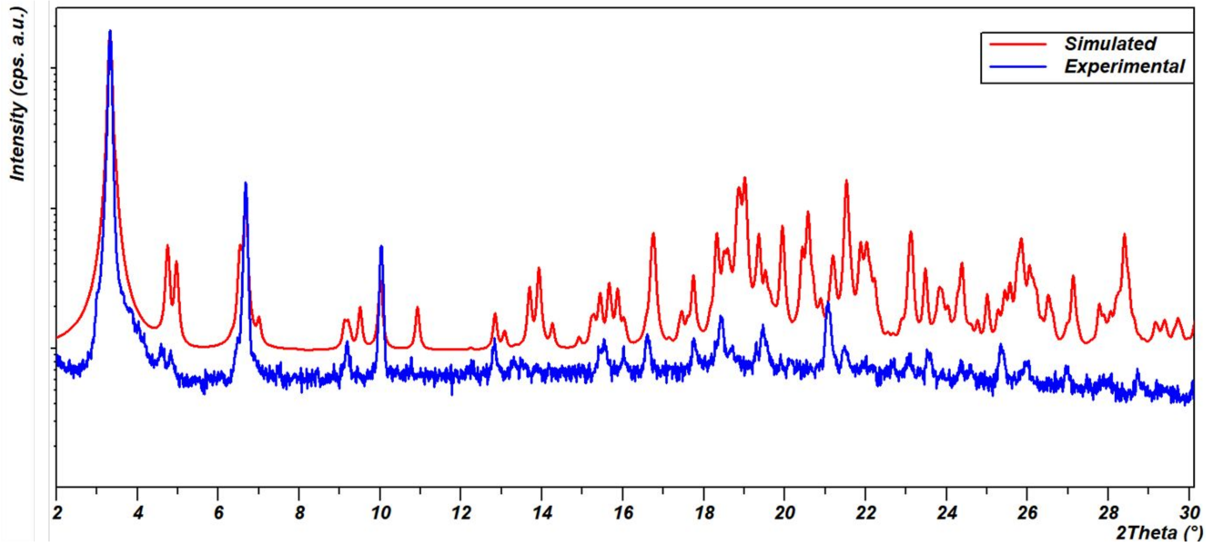

Figure S 19 Comparison and matching of experimental and simulated PXRD patterns of Form II crystal at room temperature.

Issues encountered during single-crystal X-ray diffraction (SCXRD) experiments

Crystal Data Collection: Despite multiple attempts, the crystal data collected for both Form Ia and Form II were not ideal. Both crystals were small and fragile and the manipulation inevitably led to the formation of cracks.

Form Ia: Crystal data for Form Ia were solved and refined by keeping all atoms isotropic to avoid encountering non-positive definite results. This suggests that the crystal quality was particularly poor, leading to difficulties in obtaining accurate structural information.

Form Ia Stability: Crystals of Form Ia were observed to be drastically metastable over time and eventually disappeared, making it impossible to attempt to grow better-quality crystals. This instability prevented further attempts to improve crystal quality through additional growth attempts or optimization of crystallization conditions.

Form II: Crystals of Form II exhibited a thin, needle-like morphology and demonstrated flexibility, making them susceptible to damage during handling and data collection. These characteristics likely contributed to the difficulties encountered in obtaining high-quality diffraction data for Form II.

Cell parameters of Form Ia, collected at low temperature (273K)

a= 11.8863(7) Å
b= 11.8546(7) Å
c= 43.076(3) Å
α= 90˚
β= 91.221˚(5)
γ= 90˚
Volume= 6068.4(6) Å^3
Z/Z' = 8/2

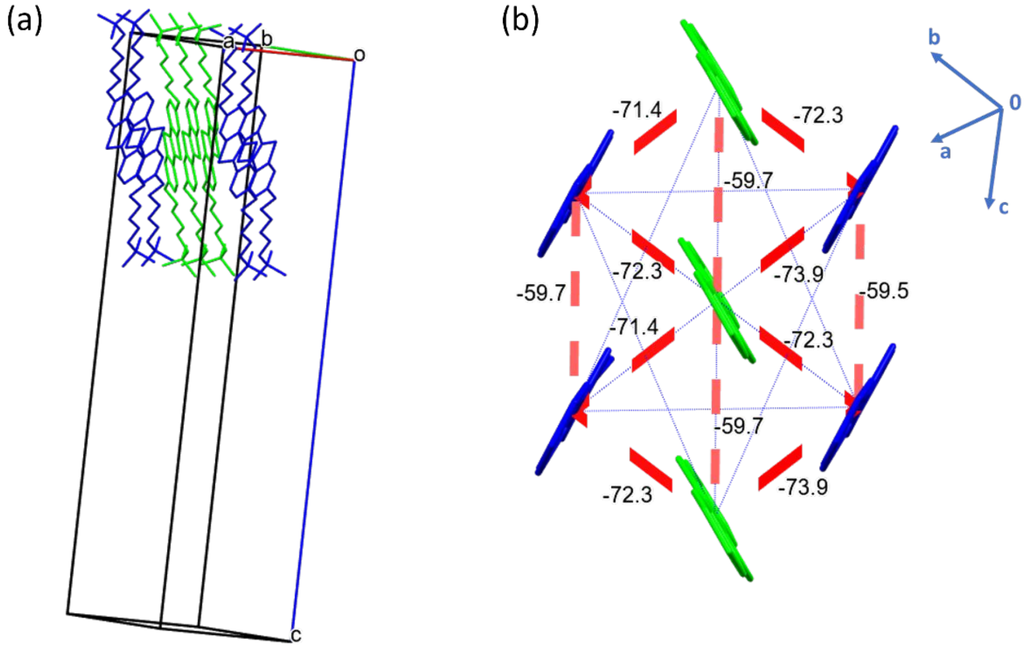

Figure S 20 Intermolecular interaction analysis for Form Ia. (a) Selected set of symmetry-equivalent molecules from the crystal packing, used for calculating pairwise interaction energies. Molecules are coloured according to symmetry equivalence. Hydrogen atoms are omitted for clarity (b) Calculated pairwise interaction energies (in kJ/mol) using Mercury software, showing the dominant interactions within the selected molecular cluster. Side chains are omitted to highlight core–core interactions.

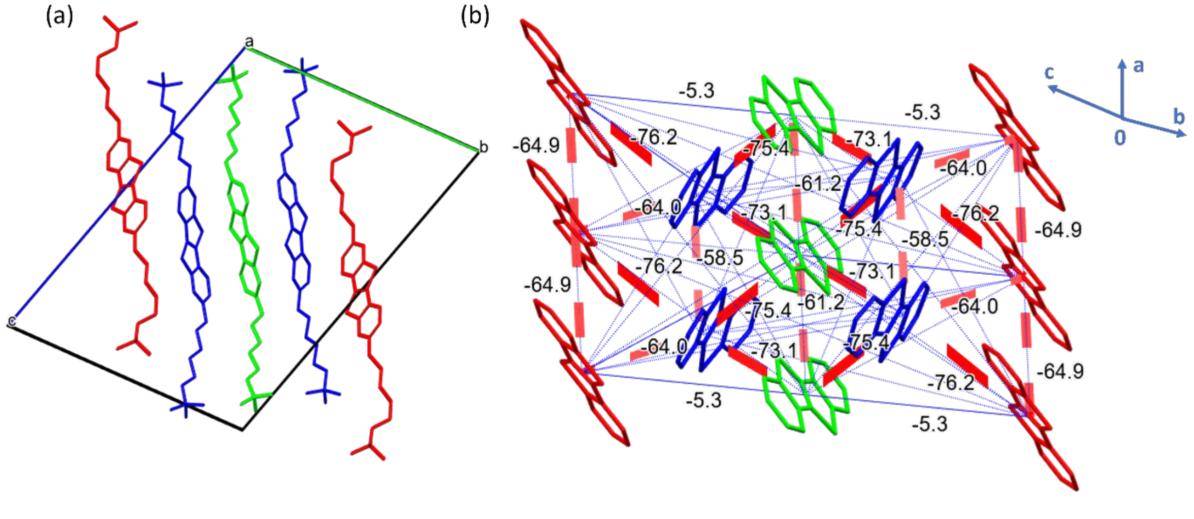

Figure S 21 Intermolecular interaction analysis for Form II. (a) Selected set of symmetry-equivalent molecules from the crystal packing, used for calculating pairwise interaction energies. Molecules are coloured according to symmetry equivalence. Hydrogen atoms are omitted for clarity (b) Calculated pairwise interaction energies (in kJ/mol) using Mercury software, showing the dominant interactions within the selected molecular cluster. Side chains are omitted to highlight core–core interactions.

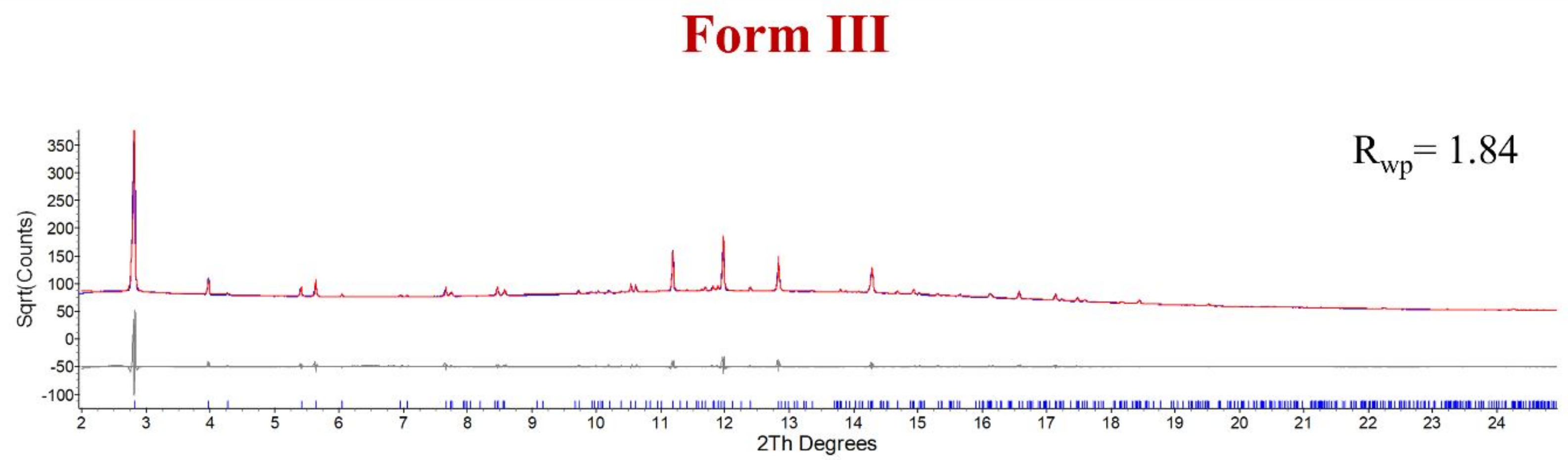

Figure S 22 Pawley refinement plot with the corresponding unit cell parameters of Form III, measured at the PSI synchrotron. Experimental XRD powder pattern (blue curve) calculated one (red curve) and difference (grey curve) are presented.

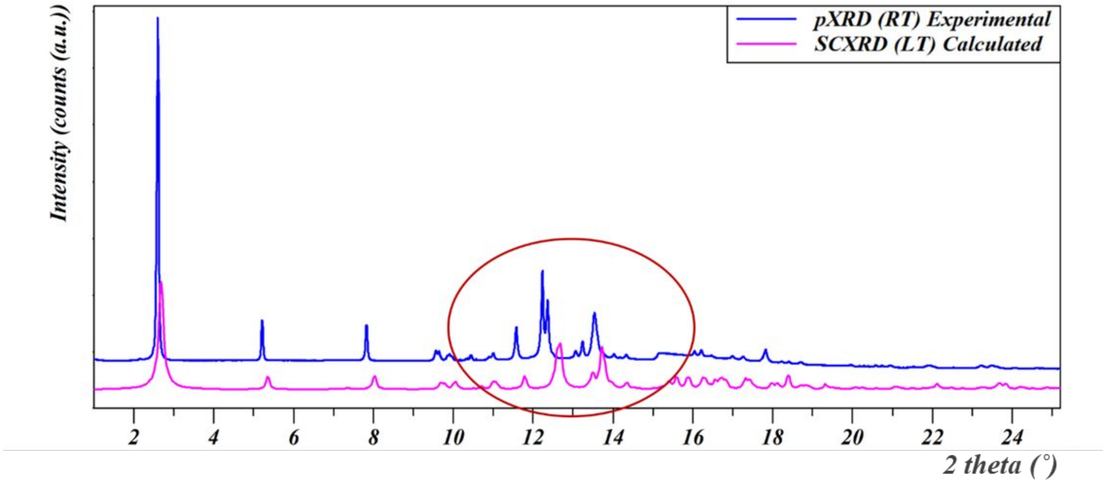

Figure S 23 Comparison of experimental (from PSI synchrotron) and simulated PXRD patterns of Form I and Form Ia, highlighting the slight difference in the patterns (top) (λ=0.9999613 Å)

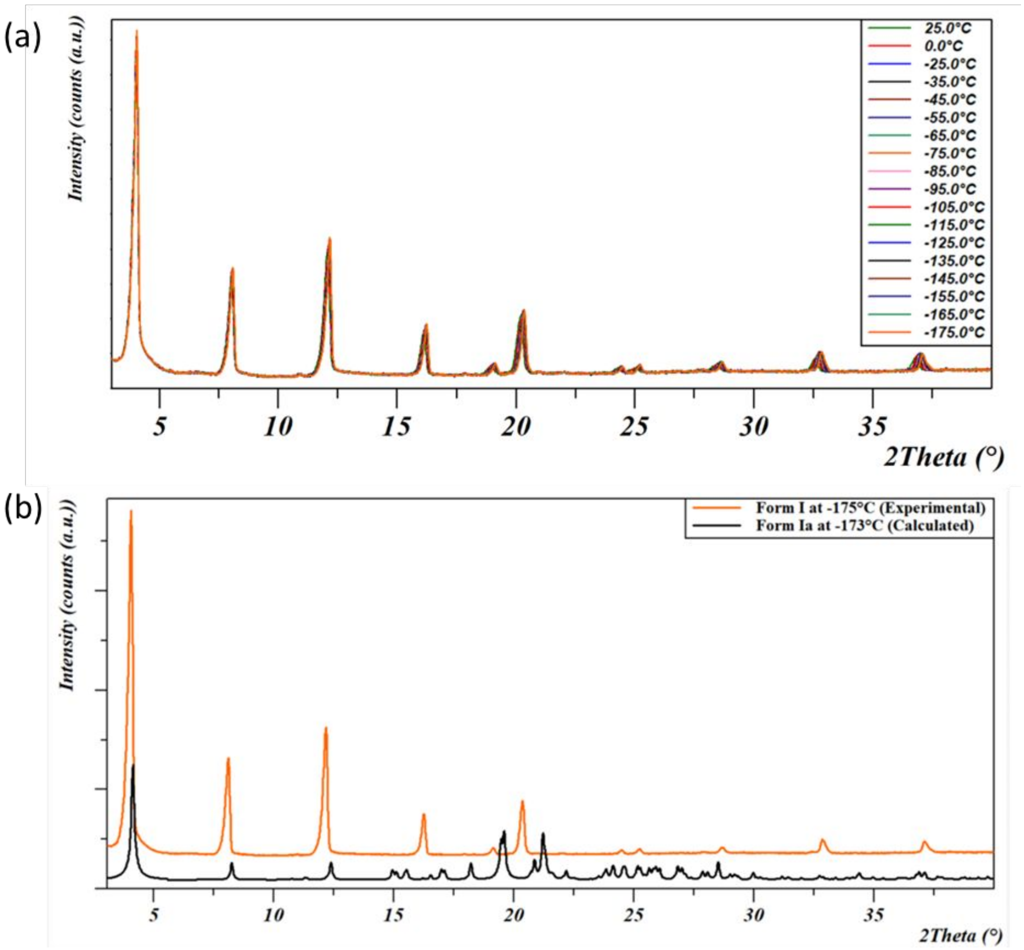

Figure S 24 (a) VTXRD of Form I (starting material) down until -175˚C and (b) comparison of experimental and calculated PXRD at low temperature. The VTXRD shows that there is neither any transition from RT to LT nor thermal contraction in Form I. (λ=1.54 Å).

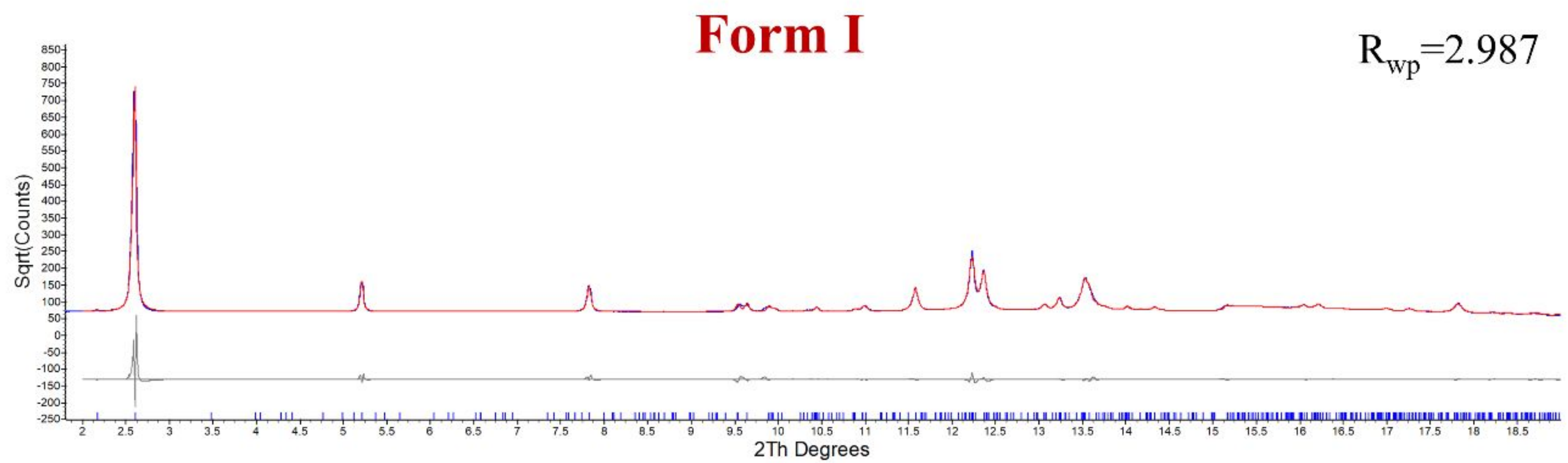

Figure S 25 Pawley refinements plot with the corresponding unit cell parameters of Form I, measured at the PSI synchrotron (λ= 0.9999613 Å). Experimental XRD powder pattern (blue curve) calculated one (red curve) and difference (grey curve) are presented.

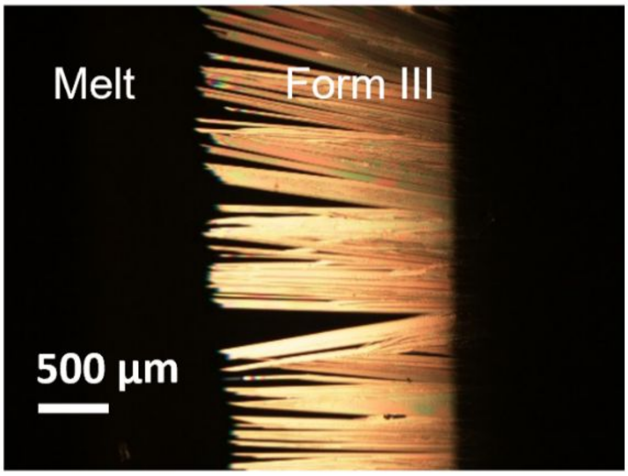

Figure S 26 In-situ POM image of Form III crystal alignment by thermal gradient technique, showing the melt and the crystal phase. T_h = 170˚C, T_c=70˚C, v= 25 µm s^{-1}.

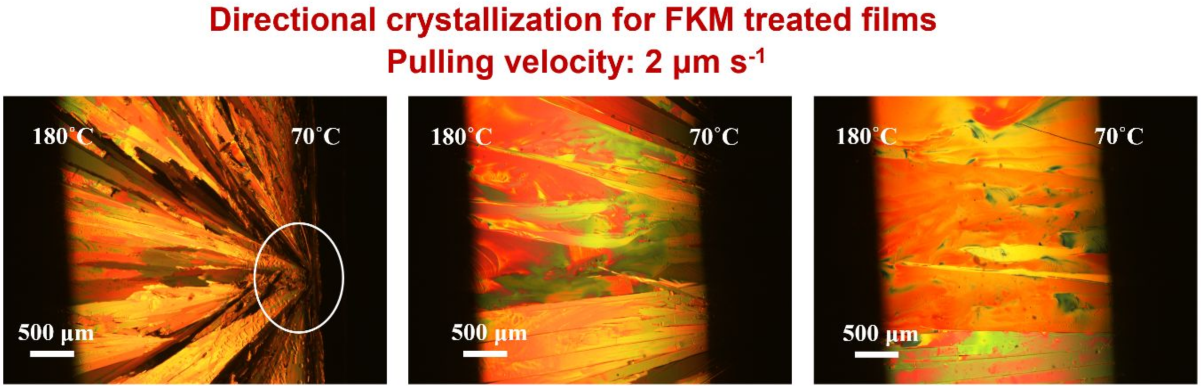

Figure S 27 Polarized Optical Microscope (POM) images at RT of directional crystallization of ditBuC6-BTBT using thermal gradient after surface treatment by FKM polymer, showing nucleation in undercooling region, alignment region and the growth region. T_h = 180˚C, T_c=70˚C, v= 2 µm s^{-1}, spin-coating of FKM by 6000 RPM and 4000 acceleration.

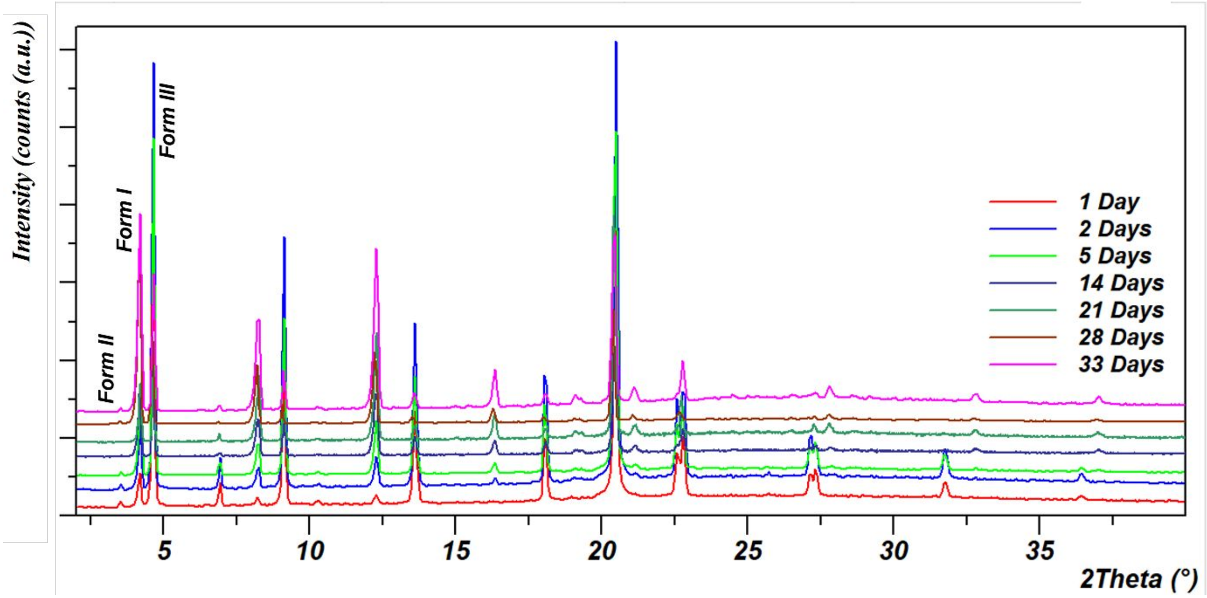

Figure S 28 PXRD pattern of films by thermal gradient representing the stability test, which reveals that Form III converts to Form I over time, which, in turn, starts to convert to Form II. However, the kinetics of transformation is very slow, and the complete conversion to Form I took more than a month time.

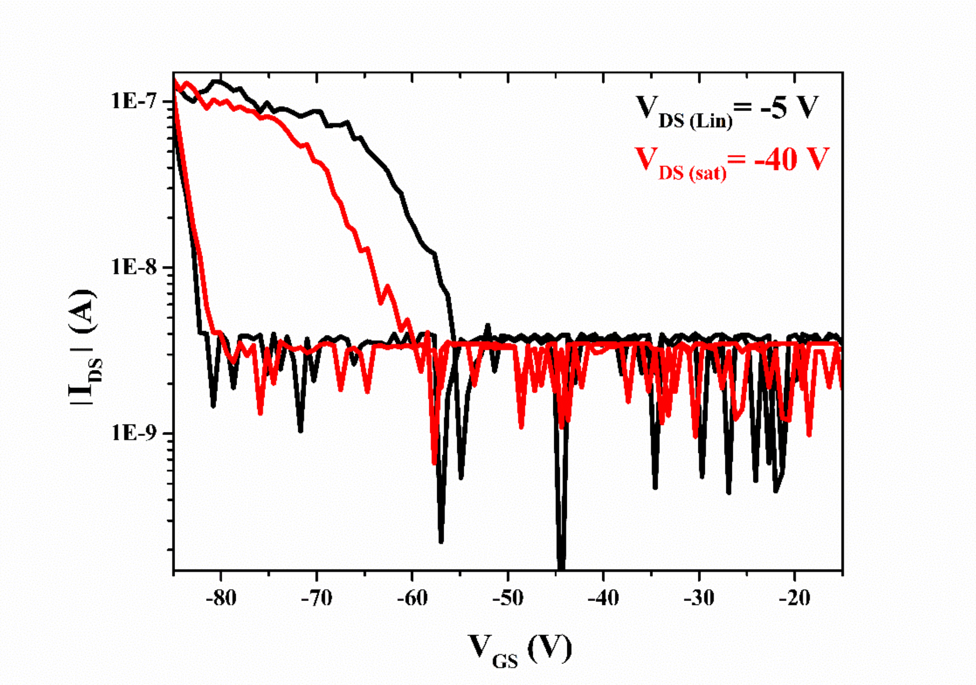

Figure S 29 Transfer characteristics of bottom gate/bottom contact (BGBC) OFET on pristine ditBuC6-BTBT deposited by solution shearing at 105˚C temperature and 10 mm s^{-1} shearing speed, showing saturation curve. W/L = 100.

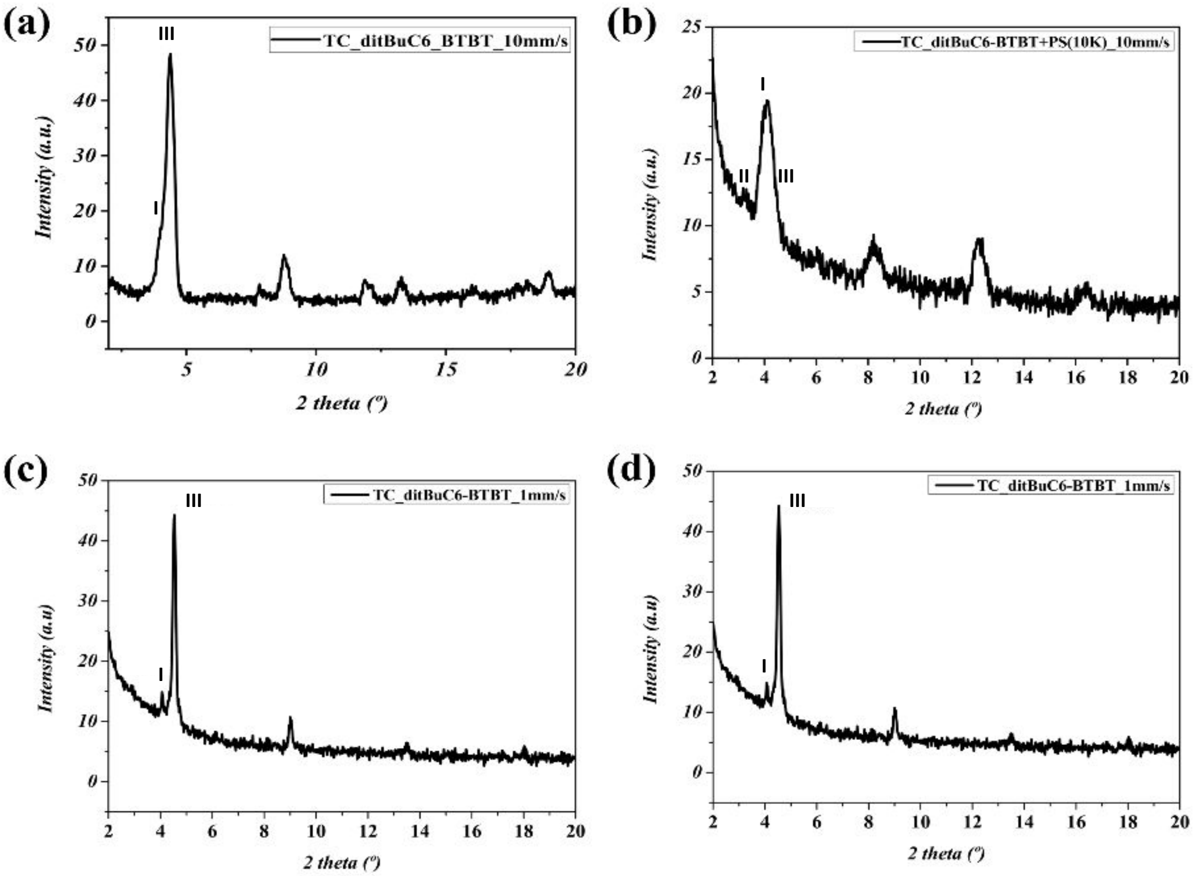

Figure S 30 XRD patterns of (a) Pristine 10 mm s^{-1} (b) ditBuC6-BTBT:PS 10 mm s^{-1}(c) Pristine 1mm s^{-1}) and (d) ditBuC6-BTBT:PS 1 mm s^{-1}. In both (a) and (b) dominant peak with the presence of a shoulder peak can be observed.

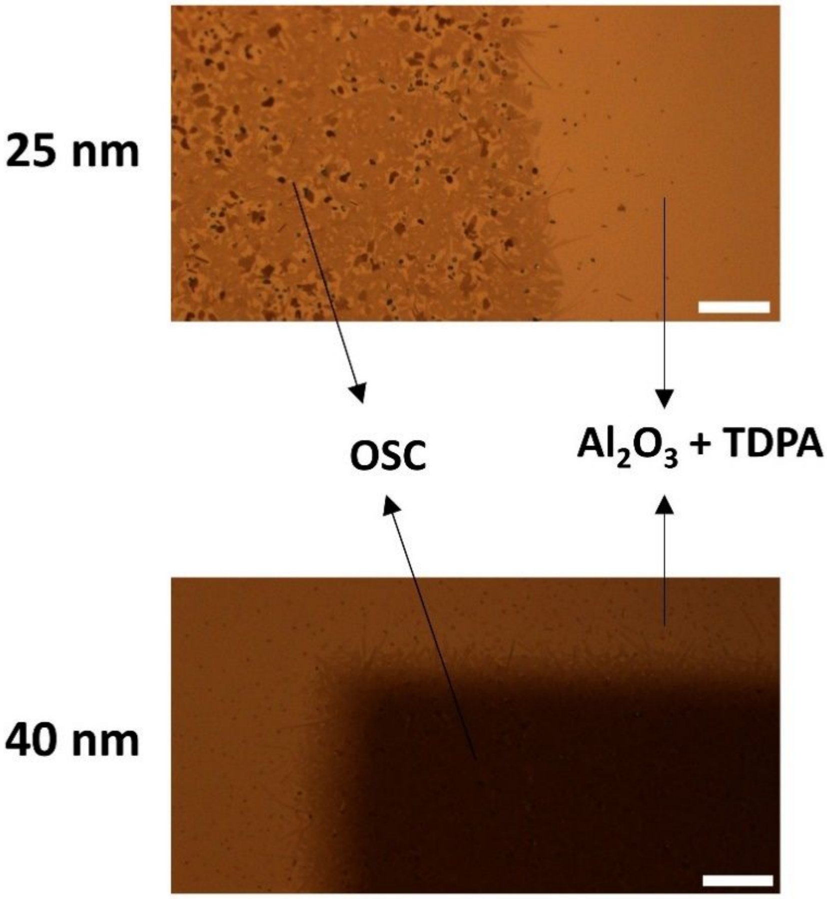

Figure S 31 Optical micrographs of ditBuC6-BTBT deposited with a nominal thickness of 25 nm (on the top) and 40 nm (on the bottom) onto Al_2O_3/TDPA at T_{sub} 40°C. Scale bar = 20 µm.

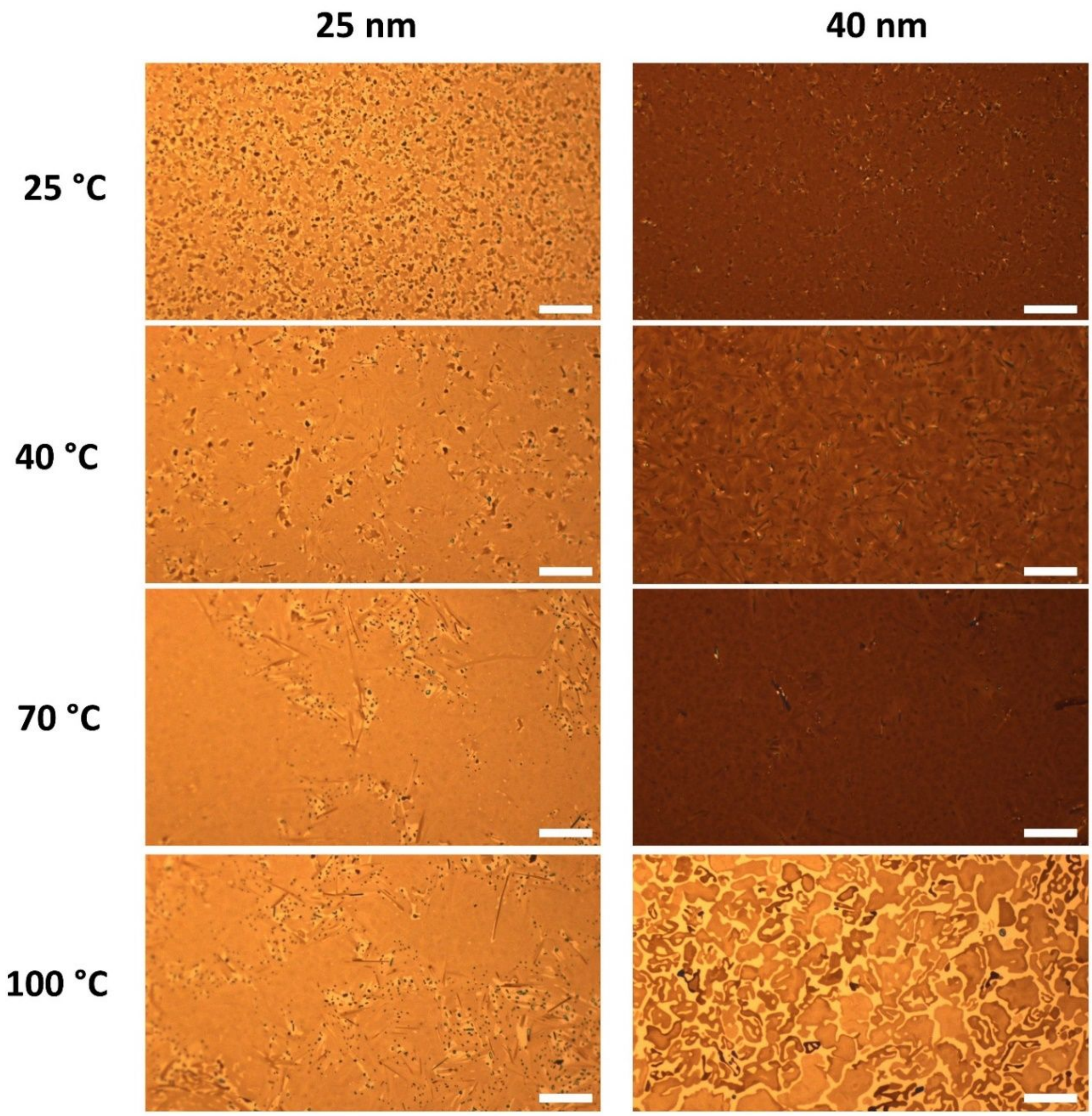

Figure S 32 Optical micrographs of ditBuC6-BTBT deposited with a nominal thickness of 25 nm (on the left) and 40 nm (on the right) onto Al_2O_3/TDPA at T_{sub} 25, 40, 70 and 100°C. Scale bar = 20 µm.
